# Sex differences in peripheral and local immune responses following spinal cord injury

**DOI:** 10.3389/fimmu.2026.1811925

**Published:** 2026-05-25

**Authors:** Sara Rito-Fernandes, Andreia Monteiro, Maria M. Moura, Juliana Fiúza-Fernandes, Sara M. Pinto, Marta F. Lima, João L. Afonso, Inês Pereira, Bárbara Carneiro-Pereira, Luís S. Fernandes, Filipa Ferreira-Antunes, Ana T. Palha, André Vidinha-Mira, António J. Salgado, Nuno A. Silva, Susana Monteiro

**Affiliations:** 1 Life and Health Sciences Research Institute (ICVS), School of Medicine, University of Minho, Braga, Portugal; 2 ICVS/3B‘s - PT Government Associate Laboratory, Braga/Guimarães, Portugal

**Keywords:** functional recovery, immune response, inflammation, sex differences, spinal cord injury

## Abstract

**Introduction:**

Biological sex has been shown to influence immune function and inflammatory responses. However, most preclinical studies in spinal cord injury (SCI) have predominantly used female animals, potentially introducing a sex bias in our understanding of post-injury inflammation. Given the central role of inflammation in secondary pathology and repair after SCI, males and females are likely to respond differently to this condition, with sex-dependent immune differences potentially shaping injury outcomes. In this study, we explored how biological sex shapes the course of inflammation after SCI and its potential impact on functional recovery.

**Methods:**

Locomotor recovery in the SCI mouse model was assessed using the Basso Mouse Scale (BMS). Autonomic recovery was analyzed through the spontaneous void spot assay. Peripheral and local immune responses were characterized by flow cytometry of blood and spinal cord samples, respectively, to track the temporal dynamics of distinct immune cell populations across acute and chronic stages of injury.

**Results:**

We found that, acutely after SCI, males exhibit higher frequencies of circulating myeloid cells, whereas females show higher numbers of these cells in the spinal cord, suggesting delayed myeloid infiltration in males. At this timepoint, females initiate an early attempt to direct some cells toward a phenotype more favorable for tissue repair, with their infiltrative monocytes expressing both pro-inflammatory and alternative activation markers. Sex differences in blood CD54 expression at 4 weeks post-injury suggest that sex-specific immune responses may continue to develop at later stages after SCI. Post-injury welfare outcomes differ between sexes, with males exhibiting delayed post-operative recovery. However, spontaneous long-term functional recovery is largely comparable between sexes after SCI.

**Conclusion:**

Overall, our data identify biological sex as a key determinant of immune and neuroinflammatory responses after SCI while suggesting a limited impact on long-term recovery. These findings highlight the lack of rationale for the underrepresentation of males in preclinical studies and emphasize the importance of sex-inclusive research to better inform personalized therapeutic strategies.

## 1 Introduction

Spinal Cord Injury (SCI) causes profound motor, sensory, and autonomic dysfunction (1), with an overactive inflammatory response contributing to extending the initial injury. Although inflammation is an integral component of the body’s response culminating in the restoration of normal function and homeostasis, following SCI, the intraspinal inflammatory response rapidly diverges from typical wound-healing dynamics. Acutely after SCI, there is a brief reparative window during which resident microglia and infiltrating innate immune cells initiate debris clearance and exhibit both pro-inflammatory and alternatively activated states that have been reported to support regeneration (2–5). However, this acute inflammatory response often fails to progress towards effective repair, as pro-inflammatory cells rapidly predominate, and their neurotoxic profiles contribute to further damage rather than repairing the primary injury (4). As inflammation becomes chronic, the sustained presence of innate phagocytic effectors (microglia, neutrophils, monocytes and macrophages), together with the recurrent infiltration of new pro-inflammatory cells and the engagement of adaptive immune amplifiers (T and B lymphocytes), creates a self-perpetuating inflammatory milieu that increasingly blurs the distinction between physiological and pathological inflammation (6–10). This disruption of the balance between pro- and anti-inflammatory immune phenotypes is a defining feature of post-SCI inflammation and a major contributor to impaired long-term neural repair.

Given the fragility of this balance after SCI, intrinsic regulators of inflammatory activity may substantially alter injury outcomes. A key determinant of inflammatory responses in health and disease is biological sex (11). Immune cells in males and females differ substantially in abundance, activation thresholds, and effector function (12). Robust evidence indicates that females generally mount stronger innate and adaptive immune responses: they express higher levels of pattern-recognition receptors such as Toll-like receptor 7 (TLR7), produce more interferon-α in response to TLR7 ligands, display enhanced phagocytic activity of neutrophils and macrophages, and show greater antigen-presentation efficiency. They also exhibit higher CD4^+^ T-cell counts and CD4/CD8 ratios, increased B cell numbers and greater antibody responses (13–22). These traits contribute to females’ lower susceptibility to infections but also to an increased severity and symptomatology when those infections occur. Conversely, males commonly exhibit weaker immune activation but higher vulnerability to systemic infections (23–25). This constellation of sex-specific immune features suggests that males and females are unlikely to respond identically to the inflammatory perturbations induced by SCI.

Understanding sex-specific immunity is particularly concerning in SCI research due to a significant discrepancy between experimental models and clinical reality. Although more than 80% of human SCI cases occur in males, most preclinical studies rely exclusively on female rodents (26, 27). This long-standing bias is often justified by reports that females exhibit superior survival rates and enhanced locomotor recovery (28–31), although the literature is inconsistent and largely restricted to motor outcomes (32, 33). As a result, key aspects of SCI biology, including immune responses, have been characterized predominantly in one sex, limiting the translational relevance of existing findings (34). Since inflammation is central to both secondary degeneration and recovery, neglecting sex as a biological variable risks obscuring critical mechanistic differences that could shape therapeutic responses.

To address this gap, we investigated the extent to which biological sex shapes the immune response following SCI, focusing on both local and peripheral immune profiles across acute and chronic phases of injury. By combining flow-cytometric profiling of blood and spinal cord immune populations with behavioral assessments of recovery, our study aimed to determine whether males and females differ in their inflammatory trajectories and whether these differences influence functional outcomes. Together, these findings provide new insights into sex-specific mechanisms of SCI pathophysiology and underscore the necessity of incorporating both sexes into immunological research in this field to guide personalized therapies.

## 2 Methods

### 2.1 Animals

All experiments were conducted in accordance with the European Directive 2010/63/EU and approved by the Animal Welfare and Ethics Review Body (ORBEA EM/ICVS-I3Bs_017-2021) and the Portuguese national authority DGAV (106397/24-S). Adult male and female C57BL/6J mice (2–3 months old; Charles River, USA) were housed at the animal facilities of the Institute of Life and Health Sciences (ICVS, Braga, Portugal) under controlled temperature (21 ± 1 °C), humidity (50–60%), and a 12 h light/dark cycle. Mice were group-housed (4–6 per cage) under sterile conditions and had ad libitum access to food (Mucedola, Italy) and water. All efforts were made to minimize animal suffering and to reduce the number of animals used.

### 2.2 Spinal cord injury surgery

Spinal cord injury surgeries were performed as previously described (35). Briefly, animals were handled every other day during the week preceding surgery to acclimate them to the experimenter’s presence and manipulation. Mice’s weight was measured one day before the procedure to calculate the appropriate anesthetic dosage. General anesthesia was induced by an intraperitoneal injection of a mixture of ketamine (Imalgene, 75 mg/kg, Merial, France) and medetomidine (Dormitor, 1 mg/kg, Pfizer, USA). Throughout induction, mice were exposed to a warm lamp to prevent body temperature from decreasing, and Vaseline was applied onto the animals’ eyes to prevent corneal damage. Adequate anesthetic depth was verified by the absence of limb withdrawal following gentle fore- and hindpaw pinches. Once anesthetized, a surgical trichotomy was performed on the animal’s back, and the area disinfected with chlorhexidine. Surgeries were performed under aseptic conditions. A dorsal midline incision was made at the level of the thoracic spine (T5-T12). The paravertebral muscles were retracted and the spinous processes and the laminar arch of T8-T9 removed, to expose the spinal cord. A 10-second compression of the spinal cord was then performed using fine forceps (Dumont #5/45° angled, FST, CA, USA), inducing a severe injury. To ensure consistency across animals, all lesions were performed using the same forceps and by the same experimenter. After injury induction, the skin was closed with 9 mm autoclips (Braintree Scientific, USA), and an intraperitoneal injection of 150 µL of atipamezole (Antisedan, Orion Corporation, Finland) was administered to revert the anesthesia. After being allowed to completely recover from the anesthesia under warm light, mice were placed in individual cages with hydrogel and moistened food pellets on the floor to facilitate access to food and water.

### 2.3 Post-operative care

Following injury and throughout all *in vivo* experiments, mice were tightly monitored and cared for. During the first seven days post-surgery, animals received twice-daily subcutaneous injections of 230 µL of a solution containing buprenorphine (Bupaq, Richer Pharma, AG, Austria) for analgesia, enrofloxacin (Baytril, Bayer, Portugal) for prophylactic antibiotherapy, vitamin supplementation (Duphalyte, Pfizer, New York, USA), and saline for fluid reposition. Manual bladder expression was performed twice per day for the first six days. Thereafter, it was carried out once per day in females, and twice per day in males, continuing until sacrifice or until spontaneous recovery of bladder control. Post-operative monitoring also included evaluation of incision healing and of recovery of general activities (including grooming, nesting and defecation), and assessment of humane endpoints using a standardized score sheet that tracks wound development, signs of autophagic behavior, and weight loss exceeding 20% of pre-surgical values. Staples were removed one week after surgery, after which animals were rehoused in groups to promote social interaction and reduce stress and anxiety.

### 2.4 Locomotor function

Locomotor performance was quantified using the BMS scale (0–9) (36). Mice were individually placed in an open-field arena for a 4-minute session, during which two independent observers, blinded to group allocation, evaluated hindlimb function according to the scale and generated a mean score across both hindlimbs. The first assessment was conducted 3–4 days post-injury as a quality control measure to exclude any partial injuries. Animals scoring higher than 2 in either hindlimb (n=1) were excluded from the study due to incomplete cord compression. Subsequent evaluations were carried out once per week for the remainder of the 30-day experimental period.

### 2.5 Autonomic function

Bladder function was evaluated by the spontaneous void spot assay, which monitors autonomic control of micturition (37). Before testing, the bladder of each mouse was manually voided, followed by a subcutaneous injection of 600 µL of saline (300 µL per hindquarter). Mice were then isolated in cages lined with Whatman paper for 4 h, without access to food or water. At the end of this period, residual bladder urine was collected and weighed, and urine spots on the Whatman paper were visualized under ultraviolet light. Voiding patterns were scored on a 0–5 scale, with a score of 5 corresponding to total bladder control. The assay was carried out at 15 and 24 dpi to ensure partial recovery of autonomic function before evaluation.

### 2.6 Flow cytometry of the blood

Flow cytometry was performed as previously described (38). Briefly, approximately 50 µL of blood was collected from the mice’s tail tips to capillary tubes with EDTA at 24 h, 2 w and 4 w post-injury. For cell staining (**Table 1**), blood samples were incubated with 50 µL of an antibody cocktail for 20 min at room temperature (RT). Erythrocytes were lysed by incubating 2 mL of Ammonium–chloride–potassium (ACK) lysis buffer for 15 min, followed by two successive washes with FACS buffer (PBS, 10% BSA, 0.1% sodium azide) to stop the reaction. Cells were then fixed with 2 mL of 2% PFA for 15 min and washed once more. Precision count beads (10 µL, Biolegend) were added to each single-cell suspension to enable calculation of the cell concentrations.

**Table 1:** Detailed list of flow cytometry antibodies.

Cell marker	Fluorochrome	Dilution	Cat#	Supplier
CD45	PercPCy5.5	1:200	103131	Biolegend, USA
CD45	PE-Cy7	1:200	103113	Biolegend, USA
CD11b	APC	1:400	101211	Biolegend, USA
CD11b	PE	1:200	101207	Biolegend, USA
Ly6G	BV650	1:200	127641	Biolegend, USA
Ly6C	BV711	1:200	128037	Biolegend, USA
CXCR1	PE	1:50	566383	BB Pharmigen, USA
CXCR2	FITC	1:100	149309	Biolegend, USA
CD62L	BV421	1:100	104435	Biolegend, USA
CD54	PE-Cy7	1:200	116121	Biolegend, USA
CCR7	BV605	1:200	120125	Biolegend, USA
CCR2	APC-Cy7	1:100	150642	Biolegend, USA
CD115	APC	1:100	135509	Biolegend, USA
CD200R	FITC	1:200	123909	Biolegend, USA
MHCII	Pacific Blue	1:200	107619	Biolegend, USA
CD16/32	BV510	1:100	156625	Biolegend, USA
CD80	BV605	1:50	104729	Biolegend, USA

To compensate for spectral overlap between the chosen fluorophores, single-stained controls were prepared using BD™ CompBeads Compensation Particles Anti-Rat/Hamster Ig, κ Set (BD Biosciences).

Data were acquired on an LSRII flow cytometer (BD, Pharminogen, California, USA), and analyzed with the FlowJo™ software (version 10.10.0, BD Biosciences). Approximately 50000 events were acquired per sample. Gating strategy is presented in **Supplementary Figure 1**. Fluorescence Minus One (FMO) controls were included to enable accurate gate placement for activation markers within each population.

### 2.7 Flow cytometry of the spinal cord

Acutely (24 h) and chronically (30 d) after injury, mice were anesthetized and transcardially perfused with 20 mL of cold Phosphate Buffered Saline (PBS) (1x) containing heparin (10 units/mL). The spinal cords were carefully isolated from the vertebral column, and a 1 cm segment centered on the lesion epicenter was excised and kept in ice-cold Dulbecco’s Modified Eagle Medium (DMEM). Single-cell suspensions were generated by mechanical dissociation in DMEM, followed by filtration through a 70 µm cell strainer to remove larger debris. The cell pellet was resuspended and centrifuged with no brake at 3500 rpm for 20 min at RT in a 37% Percoll gradient to remove myelin debris. Cells were then sequentially washed in DMEM and PBS and incubated with Zombie NIR™ viability dye (1:1000 in PBS, BioLegend) for 20 min. Surface staining was performed by incubating an antibody cocktail (**Table 1**) at RT for 15 min. Cells were fixed with 2% PFA for 15 min, washed, and resuspended in 200μl of FACS buffer. To enable cell concentration quantification, 10 µL of Precision Count beads (BioLegend) were added to each sample. For the 30-d post-injury timepoint, no viability dye was used; staining began directly with a FACS-buffer wash.

To compensate for fluorophore spectral spillover, single-stained controls were prepared using BD™ CompBeads Anti-Rat/Hamster Ig, κ Set (BD Biosciences).

Samples were acquired on an LSRII flow cytometer (BD, Pharminogen, California, USA) and subsequently processed in FlowJo™ software (v10.10.0, BD Biosciences). The gating strategy used can be found in **Supplementary Figure 2** and **3**. FMO controls were included to define gates for activation markers.

### 2.8 Statistical analysis

Sample size was estimated using G*Power software with an *a priori* approach based on a pilot experiment using BMS scoring (effect size = 0.25, α = 0.05, power = 0.8). Statistical analyses were performed using GraphPad Prism (version 8.0.1; GraphPad software, Boston, Massachusetts, USA).

Unpaired t-test was used when comparing two independent groups. Multi-factorial datasets were analyzed by two-way ANOVA followed by Tukey’s *post hoc* multiple comparisons test. Repeated measures two-way ANOVA followed by Sidak’s multiple comparisons test was used for longitudinal behavioral analyses.

Statistical significance was defined as p < 0.05 (95% confidence level), and results are presented as group mean ± standard error of the mean (SEM). Outliers were detected using the ROUT method with a Q value of 2%. Identified outliers (n = 1) were excluded from the cytometry analysis.

## 3 Results

### 3.1 Males exhibit a delayed myeloid infiltration of the spinal cord at the acute phase of SCI

**Figure 1:**
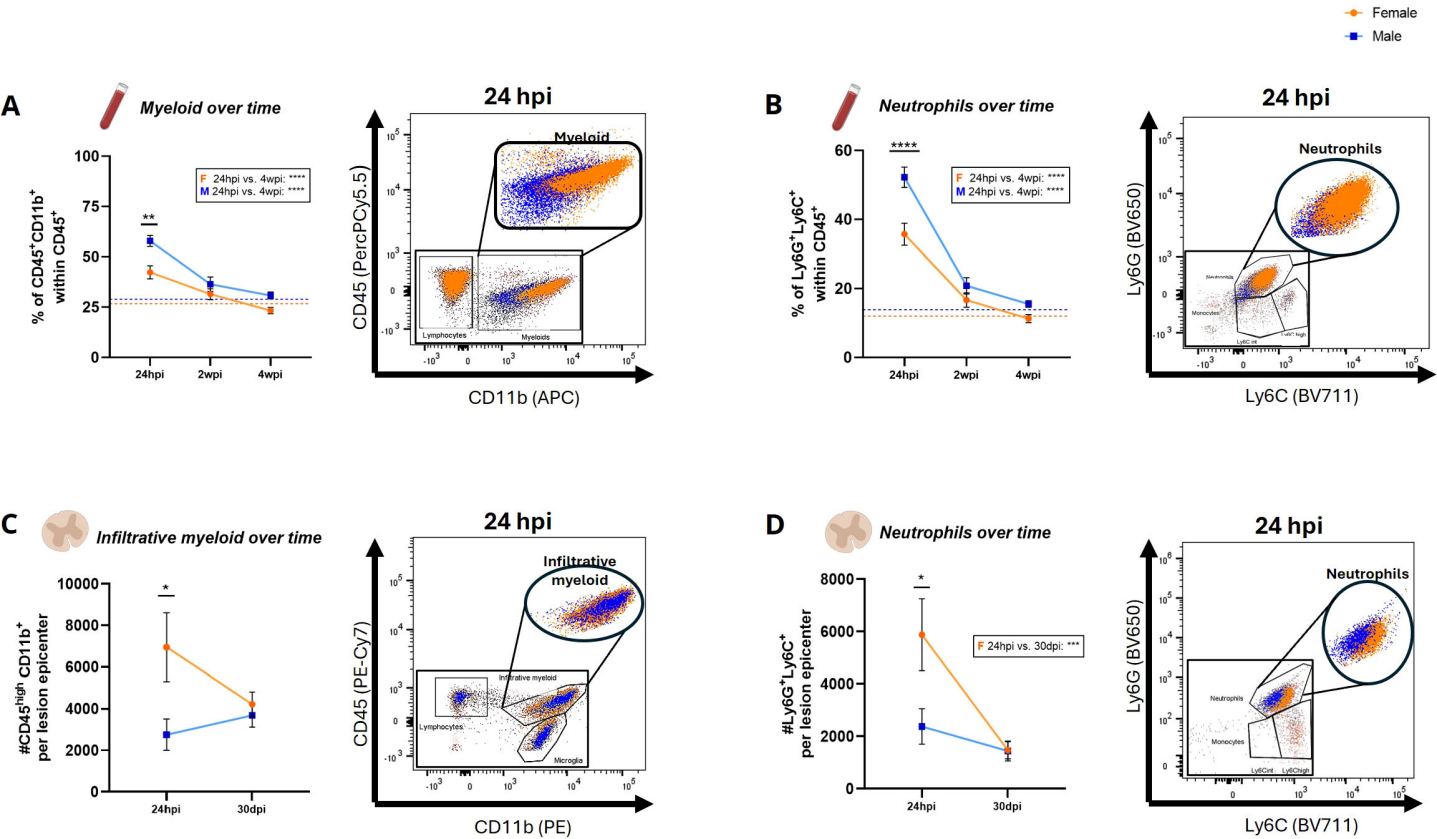
Sex-specific differences in peripheral and local inflammatory responses across acute and chronic phases of SCI. **(A, B)** Frequency of circulating **(A)** myeloid cells and **(B)** neutrophils in the blood at 24 hours, 2 weeks, and 4 weeks post-SCI in male vs. female animals, together with representative flow cytometry plots illustrating the **(A)** myeloid (CD45^+^CD11b^+^) and **(B)** neutrophil (Ly6G^+^Ly6C^+^) populations in the blood at 24 hpi. **(C, D)** Total cell counts of **(C)** infiltrative myeloid cells and **(D)** neutrophils in the spinal cord at 24 hours and 30 days post-SCI in male vs. female animals, along with representative flow cytometry plots illustrating the **(C)** infiltrative myeloid (CD45^high^CD11b^+^) and **(D)** neutrophil (Ly6G^+^Ly6C^+^) populations in the spinal cord at 24 hpi. Absolute cell numbers at the lesion epicenter were calculated using precision count beads. (Blood 24 hpi) n=13 for female, n=11 for male; (blood 2 wpi) n=13 for female, n=9 for male; (blood 4 wpi) n=11 for female, n=9 for male; (spinal cord 24 hpi) n=4 for female, n=5 for male; (spinal cord 30 dpi) n=9 for female, n=6 for male; dashed horizontal lines indicate the mean baseline values of naïve controls for each sex (orange, females; blue, males). Statistical tests: two-way ANOVA; *post-hoc* Tukey’s multiple comparisons test. Results expressed as mean ± SEM. *p ≤ 0.05, **p ≤ 0.01, ***p ≤ 0.001, ****p ≤ 0, 0001.

To investigate sex-differences in peripheral immune cell composition at different stages following SCI, we quantified the frequencies of blood leukocyte subsets at 24 hours post-injury (hpi), 2 weeks post-injury (wpi) and 4 wpi (**Figures 1A, B**, **Supplementary Figures 4A, B**). At 24 hpi, male mice exhibited higher frequencies of circulating myeloid cells than females (**Figure 1A**). Accordingly, two-way ANOVA analysis revealed a significant effect of sex on myeloid cell frequency in the blood (F (1, 60) = 17, 19; p = 0, 0001), with male animals presenting higher frequencies than females at 24 hpi (p = 0, 0012). A significant effect of time was also observed (F (2, 60) = 37, 66; p <0, 0001), driven by a reduction in myeloid cell frequencies at 4 wpi compared to 24 hpi in both males and females (p <0, 0001 for both) (**Figure 1A**). As expected, the observed pattern for the myeloid population was inverted for lymphocytes. Males displayed lower lymphocyte frequencies than females at 24 hpi (**Supplementary Figure 4A**), wich was supported by a significant effect of sex (F (1, 60) = 16, 39; p = 0, 0002; p = 0, 0017 at 24hpi). Time also significantly influenced lymphocyte frequencies (F (2, 60) = 35, 85; p <0, 0001), with both sexes presenting increased lymphocyte frequencies at 4 wpi compared with 24 hpi (p <0, 0001 for both) (**Supplementary Figure 4A**).

Analysis of myeloid subsets indicated that the sex-dependent profile in blood was largely driven by neutrophils, as no sex differences or significant effects were detected for monocytes (**Figure 1B**, **Supplementary Figure 4B**). Although time significantly affected monocyte frequencies (F (2, 60) = 26, 04; p <0, 0001), this effect did not mirror the overall myeloid profile, since both sexes exhibited increased frequencies at 4 wpi relative to 24 hpi (females: p = 0, 0165; males: p <0, 0001) (**Supplementary Figure 4B**). In contrast, neutrophil frequencies closely resembled those of the total myeloid population, showing a significant dependence on the sex of the animals (F (1, 60) = 17, 40; p <0, 0001), with males presenting higher values than females at 24 hpi (p <0, 0001). This parameter decreased over time (F (2, 60) = 93, 56; p <0, 0001), with both sexes showing lower frequencies at 4 wpi compared with 24 hpi (p <0, 0001) (**Figure 1B**).

We next compared immune cell accumulation in the spinal cord between males and females over time after SCI by quantifying infiltrating leukocytes and resident microglia at acute and chronic stages (**Figures 1C, D**, **Supplementary Figures 4C–E**). At 24 hpi, females exhibited higher numbers of infiltrating myeloid cells than males (**Figure 1C**). Accordingly, sex remained a significant factor influencing infiltrating myeloid cell counts (F (1, 20) = 7, 749; p = 0, 0115), with females exhibiting higher numbers at 24 hpi (p = 0, 0243) (**Figure 1C**). This sex-dependent profile was largely driven by neutrophils. To determine whether the marked sex-dependent differences in acute spinal cord neutrophil accumulation were also associated with altered neutrophil phenotypic features, we quantified Ly6C and Ly6G geometric mean fluorescence intensities (GMFI) within infiltrating neutrophils at 24 hpi (**Supplementary Figure 5**). While representative plots suggested an apparent broader Ly6C distribution in females (**Figure 1D**), quantitative analysis revealed no significant sex differences in Ly6C expression (nor in Ly6G), indicating that the observed differences primarily reflect neutrophil abundance rather than altered Ly6C or Ly6G-associated phenotype (**Supplementary Figure 5**). Neutrophil counts were significantly influenced by sex (F (1, 20) = 7, 791; p = 0, 0113), with females displaying higher values than males at 24 hpi (p = 0, 0106). Time also significantly influenced neutrophil numbers (F (1, 20) = 17, 89; p = 0, 0004), with females showing a decrease from 24 hpi to 30 days post-injury (dpi) (p = 0, 0004). In addition, a significant interaction between sex and time was also observed (F (1, 20) = 7, 540; p = 0, 0125) (**Figure 1D**). Monocytes also showed a trend toward a sex effect (p = 0.0666), with females tending to exhibit higher counts than males at 24 hpi (p = 0.1346) (**Supplementary Figure 4E**).

In contrast, lymphocyte and microglial populations, did not show sex-dependent differences in total cell numbers. However, time exerted a significant effect on both populations (lymphocytes: F (1, 20) = 18, 60; p = 0, 0003; microglia: F (1, 20) = 22, 79; p = 0, 0001), reflected by an increase in total cell counts overtime, which was statistically significant in females (lymphocytes: p = 0, 0039; microglia: p = 0, 0028) (**Supplementary Figures 4C, D**). Male microglia also exhibited a tendency towards decreased cell numbers from 24 hpi to 30 dpi (p = 0, 0691) (**Supplementary Figure 4D**).

### 3.2 Females combine pro-inflammatory and alternative activation profiles in the acute phase after SCI

Following the analysis of immune cell infiltration, we next assessed the activation profile of immune cells at the lesion epicenter at 24 hpi and 30 dpi, in males and females. Monocytes were analyzed for three activation markers: CD16/32, CD80, and CD200R. CD16/32 and CD80 are commonly described as pro-inflammatory activation markers, whereas CD200R can be expressed in alternatively activated myeloid cells associated with reparative profiles and resolution of inflammation (39–43).

**Figure 2:**
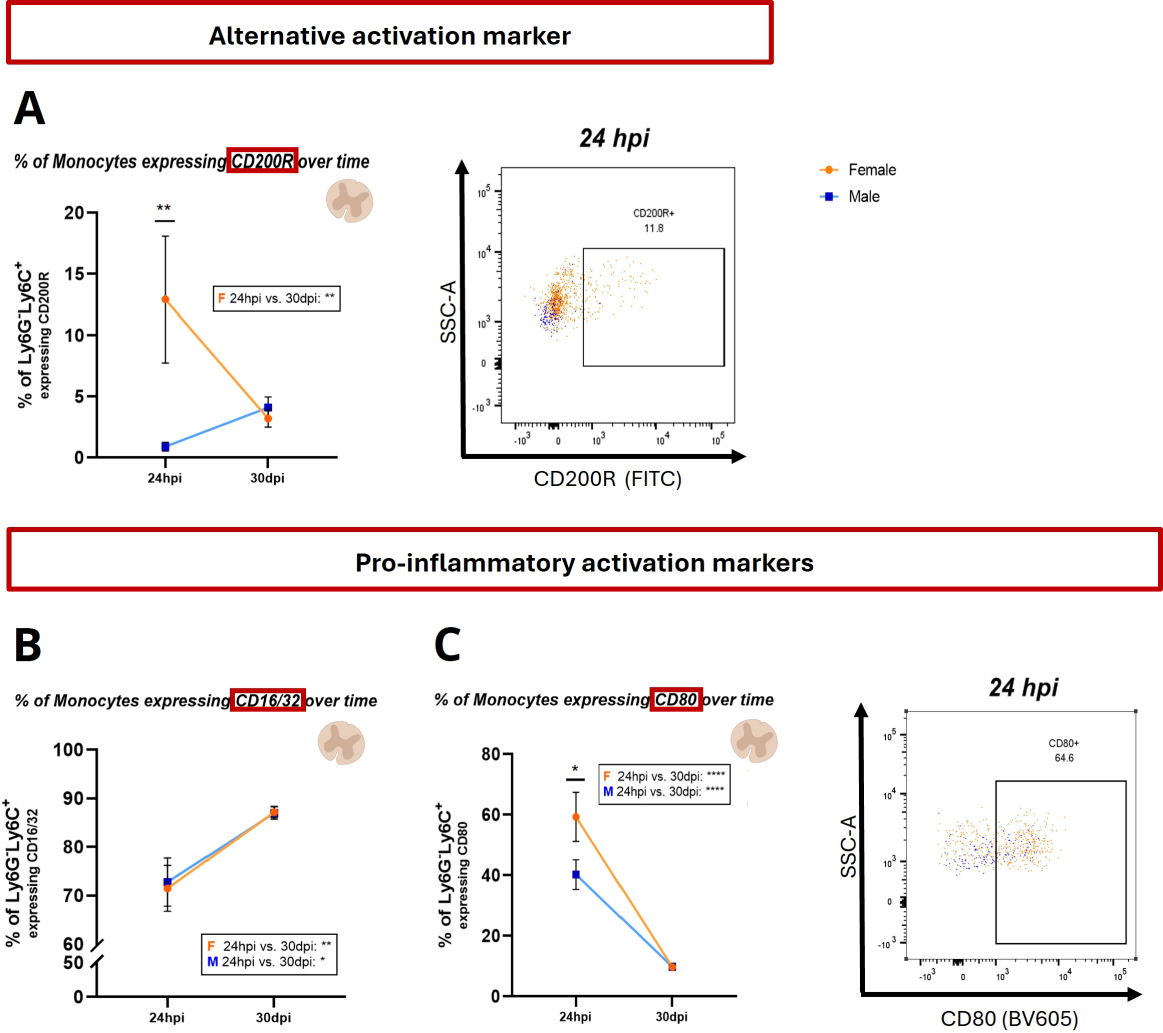
Sex-specific differences in monocyte's activation profile at the spinal cord across acute and chronic phases of SCI. **(A)** frequency of monocytes (Ly6G^-^Ly6C^+^) expressing **(A)** CD200R **(B)** CD16/32 and **(C)** CD80; at 24 hours and 30 days post-SCI, in male vs. female animals, together with representative flow cytometry plots illustrating the **(A)** CD200R^+^ monocytes and **(C)** CD80^+^ monocytes present at the spinal cord at 24 hpi. (24 hpi) n=4 for female, n=5 for male; (30 dpi) n=9 for female, n=6 for male; statistical tests: two-way ANOVA; *post-hoc* Tukey’s multiple comparisons test. Results expressed as mean ± SEM. *p ≤ 0.05, **p ≤ 0.01, ****p ≤ 0,0001.

We observed that sex significantly influenced the percentage of CD200R-expressing monocytes at 24 hpi (F (1, 20) = 8, 914; p = 0, 0073), with approximately 12% of monocytes from females expressing this marker, whereas only residual expression was detected in males (p = 0, 0028). A significant interaction between sex and time was also observed (F (1, 20) = 12, 09; p = 0, 0024), driven by a decrease in CD200R-expressing monocytes in females over time (p = 0, 0070), resulting in similarly low levels in both sexes at 30 dpi (**Figure 2A**).

For CD16/32, no sex-differences were detected in the multiple comparison *post-hoc* analysis. However, time significantly influenced the percentage of CD16/32^+^ monocytes (F (1, 20) = 27, 19; p <0, 0001), with both males and females showing an increase from 24 hpi to 30 dpi (males: p = 0, 0114; females: p = 0, 0047) (**Figure 2B**).

The percentage of CD80-expressing monocytes was also significantly affected by sex (F (1, 20) = 7, 068; p = 0, 0151), with females exhibiting higher values than males at 24 hpi (p = 0, 0140). Time additionally exerted an effect (F (1, 20) = 126, 2; p <0, 0001), with both sexes showing a decrease over time (p <0, 0001 for both). A significant interaction between sex and time was also observed (F (1, 20) = 7, 198; p = 0, 0143) (**Figure 2C**).

The percentage of microglia expressing CD200R or CD80 remained below 5% at both post-injury timepoints. Approximately 17% of microglia expressed CD16/32 across groups and timepoints; however, no significant effects of sex or time were observed. Given the absence of modulation across experimental conditions, these data are not shown.

In all other analyzed populations, only time-dependent effects were detected (F (1, 20) = 36, 06; p<0, 0001 for lymphocytes; F (1, 20) = 164, 2; p<0, 0001 for infiltrative myeloid; F (1, 20) = 62, 12; p<0, 0001 for neutrophils), characterized, similar to monocytes, by a significant reduction in CD80-expressing cells over time (**Supplementary Figure 6**).

### 3.3 Sex differences in CD54 expression emerge at later stages after SCI

To further characterize sex-dependent differences in the inflammatory profile of circulating leukocytes after SCI, we analyzed the expression of chemotaxis and adhesion-associated markers at 24 hpi, 2 wpi and 4 wpi. The molecules CD54 (ICAM-1), CD62L (L-selectin), CCR2, CCR7, CXCR1 and CXCR2, key mediators of leukocyte recruitment and migration via distinct adhesive and chemotactic pathways (44, 45), were analyzed to define temporal changes in leukocyte activation after SCI.

**Figure 3:**
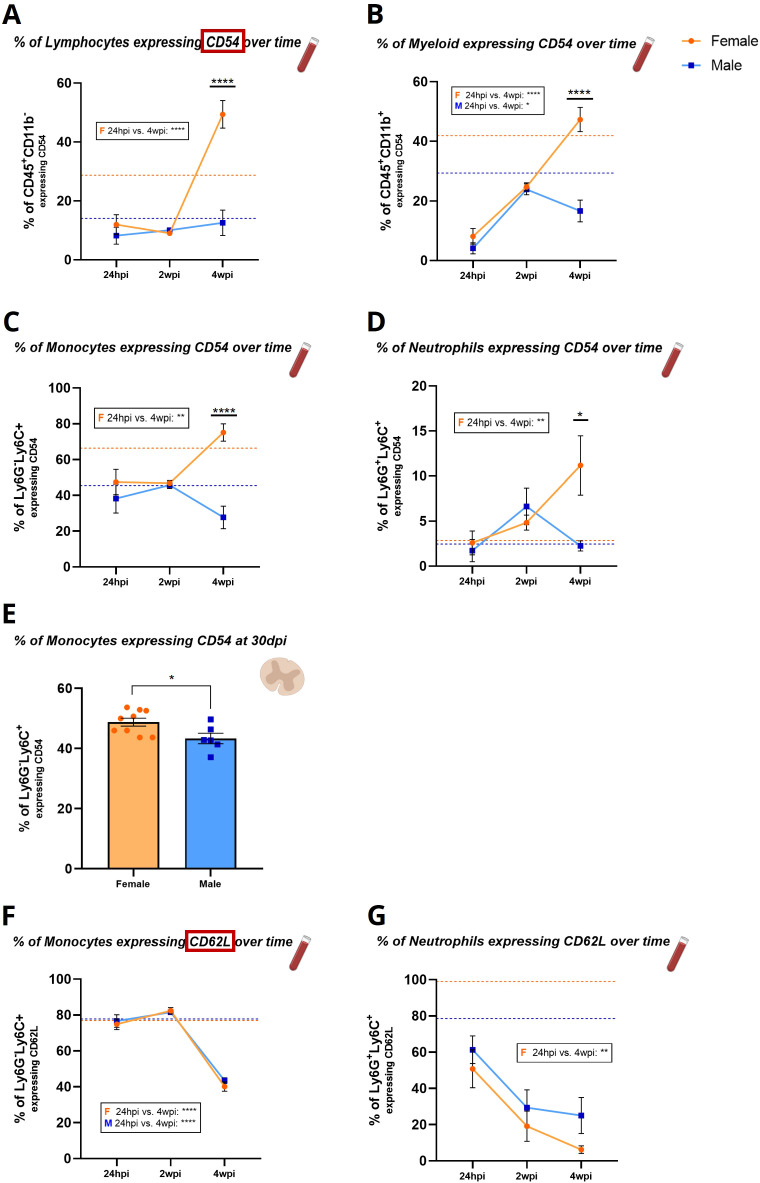
Sex-specific dynamics of cell adhesion molecules expression in circulating and infiltrative immune cells after SCI. **(A–D)** frequency of circulating **(A)** lymphocytes (CD45^+^CD11b^-^), **(B)** myeloid cells (CD45^+^CD11b^+^), **(C)** monocytes (Ly6G^-^Ly6C^+^) and **(D)** neutrophils (Ly6G^+^Ly6C^+^) expressing CD54 at 24 hours, 2 weeks and 4 weeks post-injury. **(E)** frequency of spinal cord monocytes expressing CD54 at 30 days post-injury. **(F, G)** frequency of circulating **(F)** monocytes (Ly6G^-^Ly6C^+^) and **(G)** neutrophils (Ly6G^+^Ly6C^+^) expressing CD62L at 24 hours, 2 weeks and 4 weeks post-injury. (Blood 24 hpi) n=13 for female, n=11 for male; (blood 2 wpi) n=13 for female, n=9 for male; (blood 4 wpi) n=11 for female, n=9 for male. dashed horizontal lines indicate the mean baseline values of naïve controls for each sex (orange, females; blue, males). Statistical tests: two-way ANOVA; *post-hoc* Tukey’s multiple comparisons test. (spinal cord 30 dpi) n=9 for female, n=6 for male; statistical tests: unpaired t-test. Results expressed as mean ± SEM. *p ≤ 0.05, **p ≤ 0.01, ****p ≤ 0,0001.

Sex and time both altered CD54 expression in circulating immune cells. In lymphocytes, sex (F (1, 60) = 25, 83; p <0, 0001), time (F (2, 60) = 28, 37; p <0, 0001), and their interaction (F (2, 60) = 20, 05; p <0, 0001) significantly influenced the frequency of CD54^+^ cells. Although females already showed a baseline tendency towards higher frequencies than males, females exhibited a marked increase over time (p <0, 0001), reaching 4 wpi with significantly higher frequencies of CD54^+^ lymphocytes than males (p <0, 0001) (**Figure 3A**).

A similar pattern was observed in circulating myeloid cells. Sex (F (1, 60) = 28, 29; p <0, 0001), time (F (2, 60) = 49, 16; p <0, 0001), and their interaction (F (2, 60) = 17, 25; p <0, 0001) significantly affected the percentage of myeloid cells expressing CD54. Although both sexes showed increased CD54 expression from 24 hpi to 4 wpi (males: p = 0, 0310; females: p <0, 0001), this increase was more pronounced in females, resulting in significantly higher frequencies at 4 wpi (p <0, 0001). Importantly, baseline controls revealed that females already exhibited a tendency toward higher CD54^+^ myeloid frequencies, which became further accentuated after SCI, whereas males remained below their naïve baseline levels (**Figure 3B**).

Within the monocyte subset, the frequency of CD54^+^ cells was influenced by sex (F (1, 60) = 17, 06; p = 0, 0001) and by the interaction between sex and time (F (2, 60) = 8, 953; p = 0, 0004), but not by time alone. Females displayed a progressive increase in CD54^+^ monocytes over time (p = 0, 0079), reaching significantly higher levels than males at 4 wpi (p <0, 0001) (**Figure 3C**). Similar to total myeloid cells, baseline controls indicated a pre-existing female tendency toward higher CD54 expression that became more pronounced following SCI.

In neutrophils, CD54 was expressed by a smaller proportion of cells. Nevertheless, time (F (2, 60) = 3, 757; p = 0, 0290) and the interaction between time and sex (F (2, 60) = 4, 634; p = 0, 0134) significantly influenced the percentage of CD54-expressing neutrophils. Females showed increased frequencies of CD54^+^ neutrophils at 4 wpi compared to 24 hpi (p = 0, 0086) and maintained higher levels than males at this timepoint (p =0, 0151) (**Figure 3D**). Notably, substantial inter-individual variability in CD54 expression was observed across all analyzed populations, particularly at 24 hpi and 4 wpi (**Figures 3A–D**).

To determine whether the increased frequency of circulating immune cells of females expressing CD54 at 4 wpi was also reflected at the injury site, infiltrative immune cells in the spinal cord were analyzed for the expression of this marker at 30 dpi. While no differences were detected in other immune populations (**Supplementary Figure 7**), females exhibited significantly higher CD54-expressing monocytes than males (p = 0.0267) (**Figure 3E**).

Analysis of CD62L revealed a time-dependent downregulation across myeloid populations. In circulating monocytes, time had a significant effect on the percentage of CD62L^+^ cells (F (2, 60) = 138, 3; p <0, 0001), with both males and females showing a significant decrease overtime (p <0, 0001 for both sexes) (**Figure 3F**). Similarly, the frequency of CD62L-expressing neutrophils in the blood was also significantly affected by time (F (2, 60) = 12, 47; p <0, 0001), reflected by a statistically significant decrease in females (p = 0, 0042), and a trend toward decreased values in males (p = 0, 0663) (**Figure 3G**).

**Figure 4:**
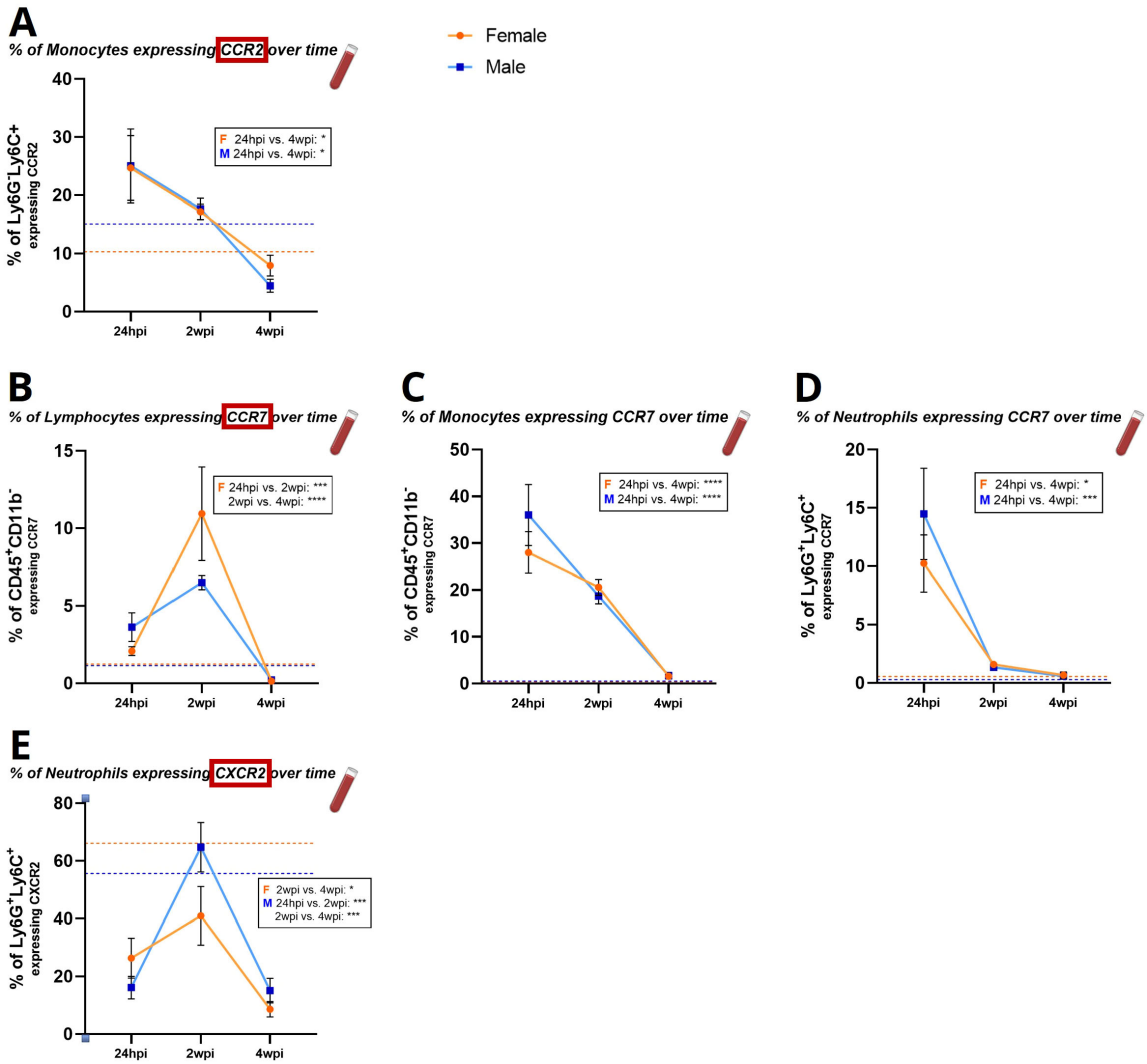
Sex-specific dynamics of chemokine receptors expression in circulating immune cells after SCI. **(A)** frequency of circulating monocytes (Ly6G^-^Ly6C^+^) expressing CCR2 at 24 hours, 2 weeks and 4 weeks post-injury. **(B–E)** frequency of circulating **(B)** lymphocytes (CD45^+^CD11b^-^), **(C)** monocytes (Ly6G^-^Ly6C^+^), and **(D)** neutrophils (Ly6G^+^Ly6C^+^) expressing CCR7, as well as **(E)** neutrophils (Ly6G^+^Ly6C^+^) expressing CXCR2, at 24 hours, 2 weeks and 4 weeks post-injury. (24 hpi) n=13 for female, n=11 for male; (2 wpi) n=13 for female, n=9 for male; (4 wpi) n=11 for female, n=9 for male. Dashed horizontal lines indicate the mean baseline values of naïve controls for each sex (orange, females; blue, males). Statistical tests: two-way ANOVA; *post-hoc* Tukey’s multiple comparisons test. Results expressed as mean ± SEM. *p ≤ 0.05, ***p ≤ 0.001, ****p ≤ 0,0001.

The frequency of CCR2^+^ circulating monocytes over time had a similar profile to that observed for CD62L^+^ cells, that progressively declined over time in both sexes, reaching residual levels by 4wpi (**Figure 4A**). This temporal reduction was supported by a significant main effect of time (F (2, 60) = 11, 13; p <0, 0001), with *post-hoc* analyses confirming decreases in both males (p = 0.0105) and females (p = 0.0305). By contrast, CCR2 expression was not detected in circulating neutrophils at measurable levels across conditions, while CXCR1 expression was minimal (<2% of neutrophils), and therefore these markers were not further pursued for neutrophil-specific comparative analyses.

The frequency of CCR7^+^ circulating immune cells also exhibits strong time-dependent regulation across subsets. In lymphocytes, CCR7^+^ cell frequencies transiently increased at 2wpi in females before declining to residual levels at 4wpi (**Figure 4B**). Accordingly, time significantly influenced the percentage of circulating lymphocytes (F (2, 60) = 15, 47; p <0, 0001), with *post-hoc* analyses confirming a transient peak of CCR7^+^ cells at 2 wpi in females (p = 0, 0005), followed by a marked decline to residual levels at 4 wpi (p <0, 0001) (**Figure 4B**).

In the monocytes, the frequency of CCR7^+^ cells was increased by SCI. However, the frequency of these CCR7^+^ monocytes decreased over time similarly in both sexes, reaching minimal levels at 4wpi (**Figure 4C**). This pattern was supported by a significant effect of time (F (2, 60) = 35, 47; p <0, 0001), with both males and females exhibiting a sustained decrease in the frequency of CCR7-expressing monocytes (p <0, 0001 for both) over time. Similarly, among assessed chemotactic receptors in neutrophils, CCR7 showed the clearest detectable temporal regulation with a time-dependent reduction in CCR7^+^ neutrophils (F (2, 60) = 21, 94; p <0, 0001), resulting in significantly lower frequencies overtime in both males and females (male: p = 0, 0003; female: p = 0, 0102), with expression becoming residual at the chronic time point (**Figure 4D**). In contrast, CXCR2^+^ neutrophils also showed significant temporal modulation (F (2, 60) = 17, 87; p <0, 0001), but with a biphasic pattern characterized by early downregulation at 24 hpi, partial recovery at 2wpi, and subsequent reduction again at 4wpi in both sexes (**Figure 4E**). *Post hoc* analysis revealed a significant increase in males from 24 hpi to 2wpi (p = 0, 0003), while females showed a similar trend. From 2wpi to 4wpi, CXCR2 expression significantly decreased in both sexes (female: p = 0, 0148; male: p = 0, 0004), indicating dynamic stage-dependent regulation of neutrophil chemotactic phenotype following SCI rather than a sustained unidirectional shift.

### 3.4 Males exhibit delayed post-operative recovery and reduced welfare after SCI

To evaluate sex differences in post-injury welfare after SCI, male and female mice were followed for 30 days post-injury and assessed for survival, body weight, and post-operative recovery parameters.

**Figure 5:**
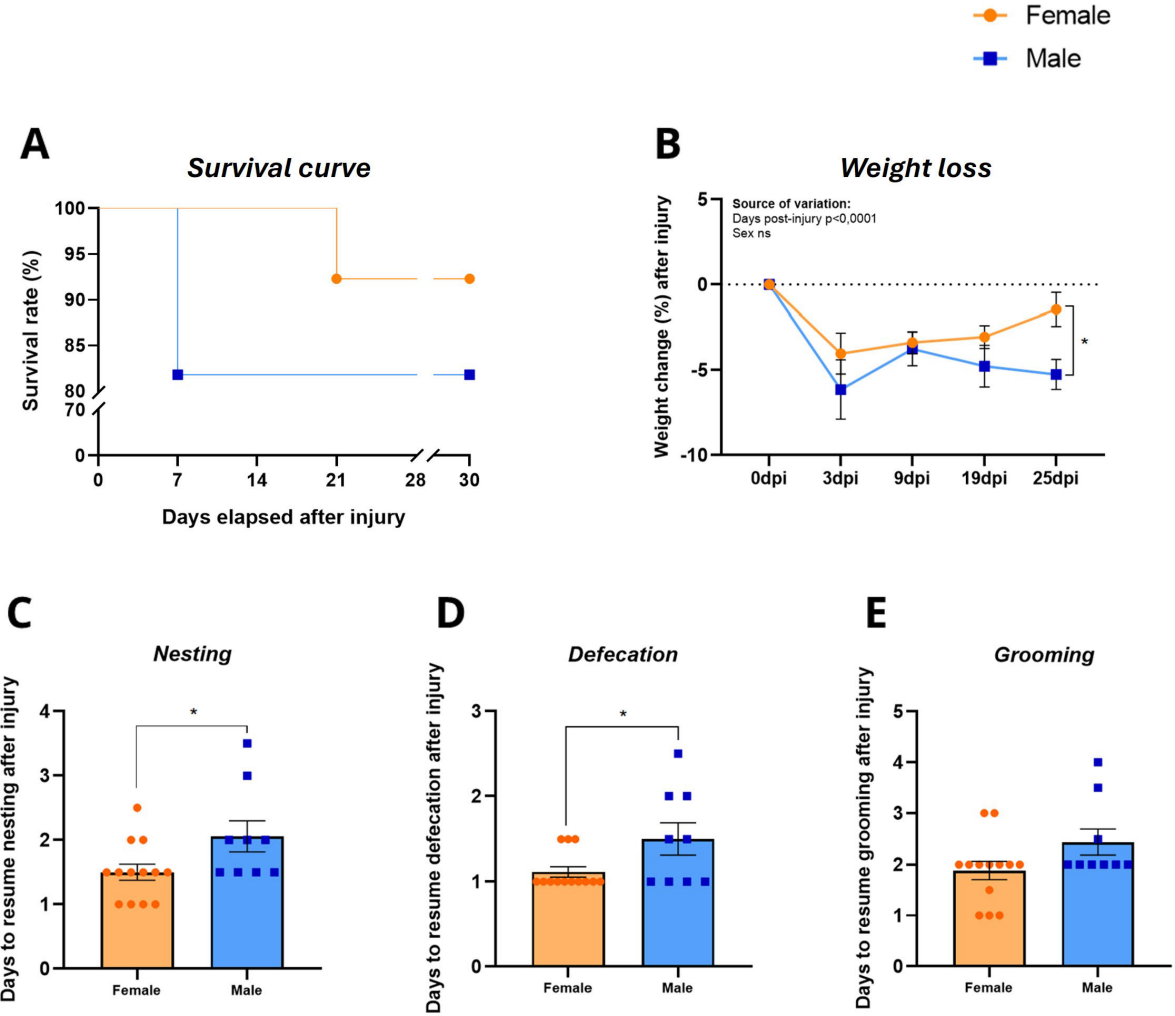
General welfare assessment after SCI. **(A)** survival rates of male and female mice throughout the 30-day *in vivo* study. **(B)** longitudinal assessment of body weight change (%) over the 30-day period. **(C–E)** number of days required for mice to resume **(C)** nesting, **(D)** defecation, and **(E)** grooming following SCI surgery. (survival curve) n=13 for female, n=11 for male; statistical tests: log-rank (Mantel-Cox) and Gehan-Breslow-Wilcoxon tests for comparison of survival curves. (weight loss) n=12 for female, n=9 for male; statistical tests: repeated measures two-way ANOVA; *post-hoc* Sidak's multiple comparisons test. (nesting, defecation, grooming) n=13 for female; n=9 for male. Statistical tests: unpaired t-test. results expressed as mean ± SEM. * p ≤ 0.05.

The study began with 24 mice (13 females and 11 males) and concluded with 21 animals (12 females and 9 males). Both male deaths occurred during the first week post-SCI and were associated with complications related to manual bladder expression. These events underscored the importance of strict bladder management in males as survival improved when bladder expression was rigorously maintained at intervals not exceeding 12 hours. The single female death occurred during the third week post-injury and resulted from the application of a humane endpoint following the development of tail necrosis. Although no statistically significant differences in survival rates were observed between sexes over the 30-day period (**Figure 5A**), the fact that male deaths resulted from similar complications, not observed in females, suggests sex-specific differences in post-injury complications.

Body weight was used as an additional indicator of animal well-being and was monitored longitudinally throughout the study. Both sexes exhibited a marked reduction in body weight at 3 dpi, followed by gradual recovery in females, although baseline values were not fully restored. In contrast, males showed partial recovery between 3 and 9 dpi but failed to maintain this trend, subsequently losing approximately 1% of body weight per week until the end of the study. By 25 dpi, the difference in weight loss between males and females became statistically significant (p = 0, 0127) (**Figure 5B**).

Post-operative recovery was further evaluated by assessing the time required for animals to resume nesting and grooming behaviors, and to initiate defecation − key indicators of general welfare (**Figures 5C–E**). Males required significantly more time to resume nesting following surgery (p = 0, 0389) (**Figure 5C**) and took significantly longer to initiate defecation compared with females (p = 0, 0350) (**Figure 5D**). No statistically significant sex differences were observed in grooming behavior (**Figure 5E**).

### 3.5 Sex does not significantly influence spontaneous functional recovery after SCI

**Figure 6:**
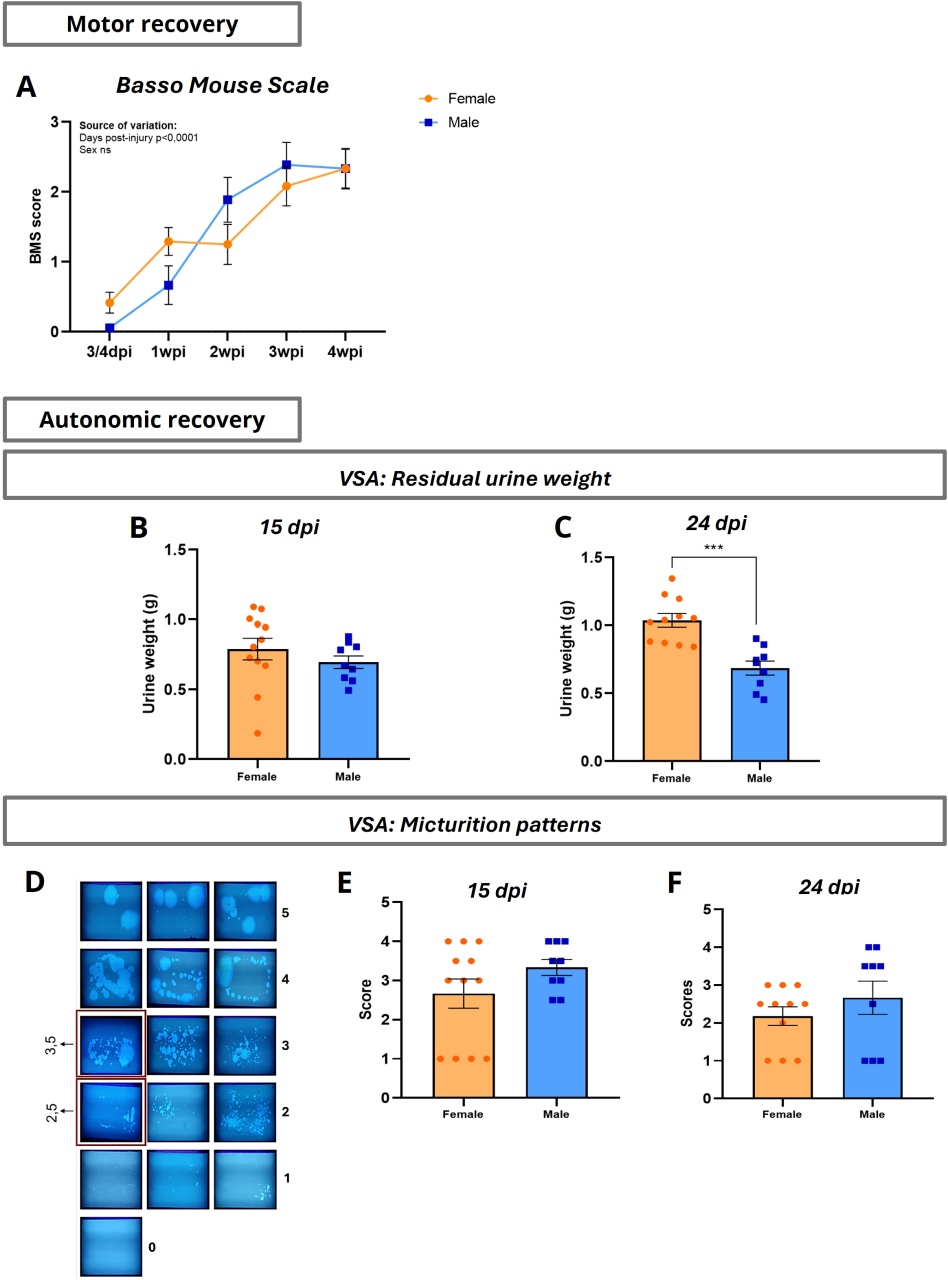
Functional recovery assessment after SCI. **(A)** locomotor recovery assessed by the BMS in male and female SCI mice over the 30-day *in vivo* study. **(B–F)** autonomic function of SCI animals assessed by void spot analysis parameters: residual urine weight (g) at the end of the 4-hours trial at **(B)** 15 dpi and **(C)** 24 dpi; and **(D)** attributed scores to urine patterns presented in the Wattman papers at the end of the 4-hours trial at **(E)** 15 dpi; and **(F)** 24 dpi. (BMS) n=12 for female, n=9 for male. Statistical tests: repeated measures two-way ANOVA; *post-hoc* Sidak's multiple comparisons test. (VSA 15 dpi) n=12 for female, n=9 for male. (VSA 24 dpi) n=11 for female, n=9 for male. Statistical tests: unpaired t-test. Results expressed as mean ± SEM. ***p ≤ 0.001. VSA, void spot assay.

To assess locomotor recovery, the BMS test was conducted at 3–4 dpi and subsequently on a weekly basis for four weeks (**Figure 6A**). Prior to injury, all mice exhibited normal locomotor function, scoring 9 points on the BMS scale. At 3–4 dpi, the BMS assessment was used to evaluate injury homogeneity and to exclude partial injuries. As expected, the mean score for both experimental groups was below 1 at this timepoint, indicating that most animals were not able to perform more than a slight ankle movement. Over the subsequent four weeks, both groups showed gradual improvements in BMS scores, consistent with some degree of spontaneous motor recovery following SCI. By the end of the study, most animals could only extensively move their hindlimbs (score of 2). Although a small number of animals exhibited slightly improved recovery, achieving scores above 3, none reached plantar stepping (score of 4). No statistically significant differences were observed between males and females at any timepoint (**Figure 6A**).

To investigate potential sex differences in bladder function, the spontaneous void spot assay was performed. Residual urine volume weighed after each bladder trial, as well as the micturition pattern scores obtained from Wattman paper analyses, were compared between groups (**Figures 6B–F**). While no statistical differences were observed at 15 dpi (**Figure 6B**), females exhibited significantly higher residual urine volumes than males at 24 dpi (p = 0, 0001) (**Figure 6C**). Although no statistically significant differences were detected between sexes in micturition pattern scores at either timepoint, notably, neither group displayed patterns comparable to those of uninjured animals (score of 5) (**Figures 6D–F**).

## 4 Discussion

Over the years, increasing attention has been given to the importance of sex as a biological variable in shaping immune responses across health and disease, with evidence showing that innate and adaptive immunity differ markedly between males and females due to genetic and hormonal influences (12). In the context of SCI, this consideration is particularly critical, as inflammation plays a central role in both secondary degeneration and recovery processes, being a major contributor to neuronal loss, glial activation, and infiltration of peripheral immune cells during secondary injury (46). Despite well-established sex differences in immune function, SCI research has historically relied predominately on female experimental models, creating a disconnect between preclinical findings and the male-biased clinical population affected by SCI (26, 27). This bias has limited our understanding of how sex-specific immune responses contribute to SCI pathophysiology and may influence therapeutic efficacy, as differences in immune recruitment, activation, and inflammatory dynamics can critically shape injury outcomes (47). Accordingly, in the present work, we systematically characterized sex differences in peripheral and local immune responses in a mouse compression model of SCI, aiming to clarify how biological sex shapes inflammatory dynamics across acute and chronic stages of injury.

In the acute phase of SCI, at 24 hpi, males exhibited a more pronounced myeloid cell response in the circulation compared to females. Interestingly, analysis of intraspinal inflammation at the same timepoint revealed an opposite trend, with males tending to present lower myeloid cell counts at the lesion site. Collectively, these findings suggest a delayed acute inflammatory response in males, characterized by an increased presence of myeloid cells in the blood, potentially reflecting delayed infiltration into the injured spinal cord. The hypothesis of a delayed infiltration on males instead of a reduced infiltration is supported by the fact that at 30 dpi there are no significant differences in the numbers of myeloid cells at the spinal cord between males and females. Furthermore, these delayed inflammatory dynamics may contribute to the slight delay observed in the recovery of general activity in males after injury.

Notably, our data indicates that the myeloid response within the spinal cord stabilizes, or even declines in the case of neutrophils, from the acute to the chronic phases. In contrast, lymphoid and microglial populations are increased at the chronic timepoint, an effect that appears to be more pronounced in females. These immune cell dynamics are consistent with the non-linear inflammatory response characteristic of SCI. The decline of neutrophils overtime aligns with their transient role during the acute phase of injury, typically arriving at the lesion site 4–6 h post-injury (48, 49), and peaking between 24 h to 3 days post-injury (48–50). The stabilization of monocyte numbers likely reflects their rapid differentiation into macrophages and associated phenotypic changes that reduce their detectability at later stages, even though infiltration peaks during subacute stages (10, 50, 51). Finally, the patterns observed for the microglial and lymphoid populations do not necessarily reflect continuous expansion. Previous studies have shown that these populations peak at intermediate stages following SCI and subsequently stabilize or partially decline (10, 52–54). Given the limited number of timepoints analyzed, it is likely that peak responses occurred at intermediate stages not captured here, with elevated cell numbers at 30 dpi reflecting persistent rather than actively expanding populations.

Regarding the activation profile of main immune populations, males presented a more pronounced pro-inflammatory profile at the acute timepoint after SCI. This was evidenced by the residual expression of CD200R, a marker associated with an alternatively activated, reparative profile, alongside robust expression of pro-inflammatory markers such as CD16/32 and CD80. In contrast, immune populations in females not only expressed pro-inflammatory markers but also showed detectable expression of CD200R, particularly within the monocyte population, suggesting that females concurrently engage pro-inflammatory and alternative activation profiles at this timepoint. This hypothesis is consistent with the broader understanding of sex-based differences in immune responses, whereby females typically mount more robust, complex and balanced immune responses than males (12). Such responses often integrate both pro-inflammatory and tissue repair mechanisms (12), which, among other things, may be associated with an enhanced sensitivity of female T-cells to interleukin-12 (IL-12) (55), a cytokine known to exert dual roles in both promoting inflammation and regulating immune responses (56, 57). CD200R, an alternative activation marker, is generally associated with reduced inflammation and improved outcomes after SCI (58). However, alternative myeloid activation has also been implicated in fibrotic processes (59). Although this is not directly linked to CD200R, we cannot exclude that the presence of these phenotypes in females may contribute to sex-dependent differences in fibrosis. Assessing this, together with fibrosis-specific markers, would be of interest in future studies.

This earlier and more coordinated immune infiltration in females may be driven, at least in part, by sex hormones, which are well-known modulators of both innate and adaptive immunity (12, 60). Estrogens have been shown to promote balanced inflammatory responses by enhancing immune cell activation while supporting reparative and pro-resolving mechanisms, whereas testosterone has been associated with delayed or attenuated immune activation (60, 61). Nevertheless, hormonal fluctuations verified during the estrous cycle represent an important limitation of the present study. Future studies controlling for estrous cycle stages could reduce variability and improve data consistency and reproducibility. Moreover, experimental approaches such as gonadectomy or controlled hormone replacement could help to directly establish a causal contribution of sex hormones to these immune differences.

Notably, despite the initial attempt at inducing some cells with an alternatively activated profile, which is more favorable for tissue repair, this reparative-associated response was no longer detectable at 30 dpi. At this chronic stage, significant levels of CD200R-expressing cells were absent even in females. This finding is consistent with the failure of inflammation resolution and the persistent, chronic nature characteristic of SCI-associated inflammation (6–10).

Overall, the sex-differences observed in the activation profile of intraspinal immune populations at 24 hpi appeared to normalize at this later timepoint, with fewer relevant effects being detected. Moreover, the expression of specific activation markers was attenuated at this chronic timepoint, as evidenced by the disappearance of CD200R-expressing cells, and the significant decrease in the percentage of cells expressing CD80 across all populations. This attenuated effect found at this later stage of SCI may help to explain the absence of significant sex-dependent effects on long-term functional recovery following injury, particularly with respect to motor outcomes. Although the bladder control trial conducted at 24 dpi revealed sex-specific differences in the residual urine volume after the testing session, no differences were detected in the qualitative analysis of micturition pattern analysis. Therefore, more comprehensive assessments of lower urinary tract function, such as cystometric and urodynamic analyses (62), would be required to determine whether sex-dependent differences exist in autonomic recovery after injury. Moreover, to assess potential sex-differences in the pathophysiological aspects of the injury that may not be reflected in the functional recovery tests, histological analysis of the spinal cord would be necessary.

Although several studies have reported sex differences in the microglia population and their activation profiles across various contexts (63, 64), including SCI (47), we did not detect sex-dependent differences in the analyzed microglial activation markers, either at acute or chronic phases after injury. However, it is important to note that many of the markers exhibited minimal expression, which limits the strength of conclusions that can be drawn from these analyses. Instead, sex-dependent effects were predominantly observed in peripheral immune compartments, particularly among circulating and infiltrating leukocytes. This compartment-specific pattern suggests that biological sex may exert a stronger influence on immune activation, priming, and trafficking in the periphery, while local cues within the injured spinal cord increasingly dominate immune behavior after infiltration. Moreover, peripheral neuroimmune circuits and the responsive nerve-associated macrophages, as the ones recently described for the sympathetic pathways innervating the spleen (65), may be more influenced by biological sex in shaping systemic inflammatory outcomes.

Leukocyte trafficking is orchestrated through distinct yet complementary mechanisms, including chemokine-driven chemotaxis and adhesion-mediated interactions with the vascular endothelium, both of which can be inferred from the expression of specific surface molecules on circulating immune cells. Chemokine receptors such as CCR2 and CCR7 primarily regulate directional migration along chemokine gradients, enabling the active recruitment of circulating immune cells during acute inflammatory responses (66–69). In contrast, cell adhesion molecules, including CD54, also known as Intercellular Adhesion Molecule 1 (ICAM-1), and CD62L, also known as L-selectin, mediate sequential steps of leukocyte–endothelium interactions, encompassing tethering, rolling, firm adhesion, and transmigration (66, 67, 69, 70). In the present study, the progressive decline in the frequencies of circulating leukocytes expressing CCR2 and CCR7 over time suggests a reduced reliance on chemokine-dependent migratory cues during the chronic phase following SCI. Notably, CD62L exhibited a similar temporal decline, yet its interpretation differs fundamentally from that of chemokine receptors. CD62L mediates early tethering and rolling of leukocytes in the circulation and is rapidly downregulated upon cellular activation through proteolytic shedding or internalization (71, 72). Therefore, the observed reduction in CD62L-expressing leukocytes likely reflects a more activated circulating phenotype rather than impaired migratory capacity. Moreover, the sustained elevation of CD54 expression at 4 wpi points to persistent activation of adhesion-related pathways in the circulation. Collectively, these findings suggest a temporal shift in the molecular mechanisms governing immune cell trafficking after SCI, characterized by diminished chemokine-dependent directional recruitment and sustained engagement of adhesion-related mechanisms at later stages after injury.

Additionally, the consistent reduction in the frequency of circulating immune cells expressing CD54 in males compared with females at 4 wpi suggests the possible emergence of sex-specific immune responses in later stages of SCI. CD54 is not only a key adhesion molecule critical for leukocyte extravasation and migration (73–75), but also functions as a ligand for Lymphocyte function-associated antigen 1 (LFA-1), thereby contributing to the regulation of adaptive T-cell responses (73, 74, 76). Notably, increased CD54 expression may reflect a primed migratory state rather than active infiltration at 30 dpi, which could explain the absence of sex-differences in spinal cord immune cell numbers at this stage. It would be valuable to investigate whether sex-differences in CD54-expressing cells contribute to differential immune responses at timepoints beyond 30 dpi. Also, the inclusion of broader immune phenotyping panels in future work would provide a more comprehensive view of sex-dependent immune states.

Taken together, our experimental design demonstrates that biological sex significantly influences immune responses, neuroinflammation, and aspects of functional recovery after injury. These findings highlight the need for sex-inclusive experimental designs and provide a framework of timing and targets for the development of personalized, sex-informed immunotherapeutic approaches for SCI.

## 5 Conclusions

This study demonstrates dynamic sex-specific differences in immune responses following SCI. Males showed delayed myeloid cell infiltration into the injured spinal cord, whereas females mounted a more complex acute response integrating pro-inflammatory and reparative features. These early immune differences were associated with delayed post-operative recovery in males but did not result in persistent sex-dependent differences in long-term motor outcomes.

Importantly, these findings highlight sex as a critical biological variable in SCI research. The context-dependent nature of immune sexual dimorphism provides no rationale for the underrepresentation of males in preclinical studies and underscores the necessity of including both sexes to accurately capture disease mechanisms and allow the development of effective, personalized therapeutic strategies after SCI.

## Data Availability

The raw data supporting the conclusions of this article will be made available by the authors, without undue reservation.

## Supplementary material

Supplementary Figure 1 Flow cytometry gating strategy for blood sample staining.

Supplementary Figure 2 Flow cytometry gating strategy for spinal cord single-cell suspension staining at 24 hpi.

Supplementary Figure 3 Flow cytometry gating strategy for spinal cord single-cell suspension staining at 30 dpi.

Supplementary Figure 4 **(A, B)** Frequency of circulating **(A)** lymphocytes and **(B)** monocytes in the blood at 24 hours, 2 weeks, and 4 weeks post-SCI in male vs. female animals. **(C–E)** Total cell counts of **(C)** Lymphocytes, **(D)** Microglia and **(E)** monocytes in the spinal cord at 24 hours and 30 days post-SCI in male vs. female animals. Absolute cell numbers at the lesion epicenter were calculated using precision count beads. (Blood 24 hpi) n=13 for Female, n=11 for Male; (Blood 2 wpi) n=13 for Female, n=9 for Male; (Blood 4 wpi) n=11 for Female, n=9 for Male; (Spinal Cord 24 hpi) n=4 for Female, n=5 for Male; (Spinal Cord 30 dpi) n=9 for Female, n=6 for Male; Statistical tests: two-way ANOVA; *post-hoc* Tukey’s multiple comparisons test. Results expressed as mean ± SEM. *p ≤ 0.05, **p ≤ 0.01, ****p ≤ 0, 0001.

Supplementary Figure 5 **(A, B)** Geometric mean fluorescence intensity (GMFI) of **(A)** Ly6C and **(B)** Ly6G expression in spinal cord-infiltrating neutrophils (Ly6G^+^Ly6C^+^) at 24 hours post-SCI in male vs. female animals. Quantification was performed at the lesion epicenter by flow cytometry. (Spinal Cord 24 hpi) n=4 for Female, n=5 for Male. Statistical tests: unpaired t-test. Results expressed as mean ± SEM.

Supplementary Figure 6 **(A–C)** Frequency of **(A)** lymphocytes, **(B)** infiltrative myeloid, and **(C)** neutrophils expressing CD80 in the spinal cord at 24 hours and 30 days post-SCI in male vs. female animals. (Spinal Cord 24 hpi) n=4 for Female, n=5 for Male; (Spinal Cord 30 dpi) n=9 for Female, n=6 for Male; Statistical tests: two-way ANOVA; *post-hoc* Tukey’s multiple comparisons test. Results expressed as mean ± SEM. **p ≤ 0.01, ***p ≤ 0.001, ****p ≤ 0, 0001.

Supplementary Figure 7 **(A–D)** Frequency of **(A)** lymphocytes, **(B)** microglia, **(C)** infiltrative myeloid, and **(D)** neutrophils expressing CD54 in the spinal cord at 30 days post-SCI in male vs. female animals. n=9 for Female, n=6 for Male; Statistical tests: Unpaired t-test. Results expressed as mean ± SEM.

## References

[1] Moura MM Monteiro A Salgado AJ Silva NA Monteiro S . Disrupted autonomic pathways in spinal cord injury: Implications for the immune regulation. Neurobiol Dis. (2024) 195:106500. doi: 10.1016/j.nbd.2024.106500. PMID: 38614275

[2] Fouad K Krajacic A Tetzlaff W . Spinal cord injury and plasticity: Opportunities and challenges. Brain Res Bull. (2011) 84:337–42. doi: 10.1016/j.brainresbull.2010.04.017. PMID: 20471456

[3] Oyinbo C . Secondary injury mechanisms in traumatic spinal cord injury: A nugget of this multiply cascade. tr. (2011) 71:281–99. doi: 10.55782/ane-2011-1848. PMID: 21731081

[4] Monteiro S Salgado AJ Silva NA . Immunomodulation as a neuroprotective strategy after spinal cord injury. Neural Regener Res. (2018) 13:423–4. doi: 10.4103/1673-5374.228722. PMID: 29623924 PMC5900502

[5] Stirling DP Yong VW . Dynamics of the inflammatory response after murine spinal cord injury revealed by flow cytometry. J Neurosci Res. (2008) 86:1944–58. doi: 10.1002/jnr.21659. PMID: 18438914

[6] Bareyre FM Schwab ME . Inflammation, degeneration and regeneration in the injured spinal cord: Insights from DNA microarrays. Trends Neurosci. (2003) 26:555–63. doi: 10.1016/j.tins.2003.08.004. PMID: 14522149

[7] Bethea JR . Spinal cord injury-induced inflammation: A dual-edged sword. Prog Brain Res. (2000) 128:33–42. doi: 10.1016/S0079-6123(00)28005-9. PMID: 11105667

[8] Hausmann ON . Post-traumatic inflammation following spinal cord injury. Spinal Cord. (2003) 41:369–78. doi: 10.1038/sj.sc.3101483. PMID: 12815368

[9] Popovich PG Jones TB . Manipulating neuroinflammatory reactions in the injured spinal cord: Back to basics. Trends Pharmacol Sci. (2003) 24:13–7. doi: 10.1016/S0165-6147(02)00006-8. PMID: 12498725

[10] Trivedi A Olivas AD Noble-Haeusslein LJ . Inflammation and spinal cord injury: Infiltrating leukocytes as determinants of injury and repair processes. Clin Neurosci Res. (2006) 6:283–92. doi: 10.1016/j.cnr.2006.09.007. PMID: 18059979 PMC1864937

[11] Rito-Fernandes S Salgado AJ Silva NA Monteiro S . Spinal cord injury–associated disruption of the autonomic immune control: Does biological sex matter? Neural Regener Res. (2026) 21:298. doi: 10.4103/NRR.NRR-D-24-01078. PMID: 39851133 PMC12094543

[12] Klein SL Flanagan KL . Sex differences in immune responses. Nat Rev Immunol. (2016) 16:626–38. doi: 10.1038/nri.2016.90. PMID: 27546235

[13] Abdullah M Chai PS Chong MY Tohit ERM Ramasamy R Pei CP . Gender effect on *in vitro* lymphocyte subset levels of healthy individuals. Cell Immunol. (2012) 272:214–9. doi: 10.1016/j.cellimm.2011.10.009. PMID: 22078320

[14] Berghöfer B Frommer T Haley G Fink L Bein G Hackstein H . TLR7 ligands induce higher IFN-alpha production in females. J Immunol. (2006) 177:2088–96. doi: 10.4049/jimmunol.177.4.2088. PMID: 16887967

[15] Furman D Hejblum BP Simon N Jojic V Dekker CL Thiébaut R . Systems analysis of sex differences reveals an immunosuppressive role for testosterone in the response to influenza vaccination. Proc Natl Acad Sci USA. (2014) 111:869–74. doi: 10.1073/pnas.1321060111. PMID: 24367114 PMC3896147

[16] Lee BW Yap HK Chew FT Quah TC Prabhakaran K Chan GS . Age- and sex-related changes in lymphocyte subpopulations of healthy Asian subjects: From birth to adulthood. Cytometry. (1996) 26:8–15. doi: 10.1002/(SICI)1097-0320(19960315)26:1<8::AID-CYTO2>3.0.CO;2-E. PMID: 8809475

[17] Lisse IM Aaby P Whittle H Jensen H Engelmann M Christensen LB . T-lymphocyte subsets in West African children: Impact of age, sex, and season. J Pediatr. (1997) 130:77–85. doi: 10.1016/s0022-3476(97)70313-5. PMID: 9003854

[18] Pisitkun P Deane JA Difilippantonio MJ Tarasenko T Satterthwaite AB Bolland S . Autoreactive B cell responses to RNA-related antigens due to TLR7 gene duplication. Science. (2006) 312:1669–72. doi: 10.1126/science.1124978. PMID: 16709748

[19] Spitzer JA . Gender differences in some host defense mechanisms. Lupus. (1999) 8:380–3. doi: 10.1177/096120339900800510. PMID: 10455517

[20] Teixeira D Longo-Maugeri IM Santos JLF Duarte YAO Lebrão ML Bueno V . Evaluation of lymphocyte levels in a random sample of 218 elderly individuals from São Paulo city. Rev Bras Hematol Hemoter. (2011) 33:367–71. doi: 10.5581/1516-8484.20110100. PMID: 23049341 PMC3415787

[21] Uppal SS Verma S Dhot PS . Normal values of CD4 and CD8 lymphocyte subsets in healthy Indian adults and the effects of sex, age, ethnicity, and smoking. Cytometry B Clin Cytom. (2003) 52:32–6. doi: 10.1002/cyto.b.10011. PMID: 12599179

[22] Weinstein Y Ran S Segal S . Sex-associated differences in the regulation of immune responses controlled by the MHC of the mouse. J Immunol. (1984) 132:656–61. doi: 10.4049/jimmunol.132.2.656. PMID: 6228595

[23] Klein SL Roberts C eds. Sex hormones and immunity to infection. Berlin, Heidelberg: Springer (2010). Available online at: https://link.springer.com/10.1007/978-3-642-02155–8. doi: 10.1007/978-3-642-02155-8

[24] Klein SL Roberts CW eds. Sex and gender differences in infection and treatments for infectious diseases. Cham: Springer International Publishing (2015). Available online at: https://link.springer.com/10.1007/978-3-319-16438–0. doi: 10.1007/978-3-319-16438-0

[25] Steeg L Klein SL . SeXX matters in infectious disease pathogenesis. PloS Pathog. (2016) 12:e1005374. doi: 10.1371/journal.ppat.1005374. PMID: 26891052 PMC4759457

[26] Ahuja CS Wilson JR Nori S Kotter MRN Druschel C Curt A . Traumatic spinal cord injury. Nat Rev Dis Primers. (2017) 3:17018. doi: 10.1038/nrdp.2017.18. PMID: 28447605

[27] Zizzo J Gater DR Hough S Ibrahim E . Sexuality, intimacy, and reproductive health after spinal cord injury. J Pers Med. (2022) 12:1985. doi: 10.3390/jpm12121985. PMID: 36556205 PMC9781084

[28] Datto JP Bastidas JC Miller NL Shah AK Arheart KL Marcillo AE . Female rats demonstrate improved locomotor recovery and greater preservation of white and gray matter after traumatic spinal cord injury compared to males. J Neurotrauma. (2015) 32:1146–57. doi: 10.1089/neu.2014.3702. PMID: 25715192 PMC4507304

[29] Farooque M Suo Z Arnold PM Wulser MJ Chou CT Vancura RW . Gender-related differences in recovery of locomotor function after spinal cord injury in mice. Spinal Cord. (2006) 44:182–7. doi: 10.1038/sj.sc.3101816. PMID: 16130019

[30] Ghnenis AB Burns DT Osimanjiang W He G Bushman JS . A long-term pilot study on sex and spinal cord injury shows sexual dimorphism in functional recovery and cardio-metabolic responses. Sci Rep. (2020) 10:2762. doi: 10.1038/s41598-020-59628-6. PMID: 32066802 PMC7026076

[31] Xiao J Zhang J Zhao Y Huang W Guo Z Su B . Sex differences of steroid receptor coactivator-1 expression after spinal cord injury in mice. Neurol Res. (2017) 39:1022–7. doi: 10.1080/01616412.2017.1367077. PMID: 28816099

[32] Fukutoku T Kumagai G Fujita T Sasaki A Wada K Liu X . Sex-related differences in anxiety and functional recovery after spinal cord injury in mice. J Neurotrauma. (2020) 37:2235–43. doi: 10.1089/neu.2019.6929. PMID: 32486893

[33] Walker CL Fry CME Wang J Du X Zuzzio K Liu NK . Functional and histological gender comparison of age-matched rats after moderate thoracic contusive spinal cord injury. J Neurotrauma. (2019) 36:1974–84. doi: 10.1089/neu.2018.6233. PMID: 30489213 PMC6599384

[34] Clayton JA Collins FS . Policy: NIH to balance sex in cell and animal studies. Nature. (2014) 509:282–3. doi: 10.1038/509282a. PMID: 24834516 PMC5101948

[35] Pinho AG Cibrão JR Lima R Gomes ED Serra SC Lentilhas-Graça J . Immunomodulatory and regenerative effects of the full and fractioned adipose tissue derived stem cells secretome in spinal cord injury. Exp Neurol. (2022) 351:113989. doi: 10.1016/j.expneurol.2022.113989. PMID: 35065953

[36] Basso DM Fisher LC Anderson AJ Jakeman LB Mctigue DM Popovich PG . Basso Mouse Scale for locomotion detects differences in recovery after spinal cord injury in five common mouse strains. J Neurotrauma. (2006) 23:635–59. doi: 10.1089/neu.2006.23.635. PMID: 16689667

[37] Hill WG Zeidel ML Bjorling DE Vezina CM . Void spot assay: Recommendations on the use of a simple micturition assay for mice. Am J Physiol Renal Physiol. (2018) 315:F1422–9. doi: 10.1152/ajprenal.00350.2018. PMID: 30156116 PMC6293303

[38] Monteiro S Pinho AG Macieira M Serre-Miranda C Cibrão JR Lima R . Splenic sympathetic signaling contributes to acute neutrophil infiltration of the injured spinal cord. J Neuroinflamm. (2020) 17:282. doi: 10.1186/s12974-020-01945-8. PMID: 32967684 PMC7513542

[39] Dash SP Gupta S Sarangi PP . Monocytes and macrophages: Origin, homing, differentiation, and functionality during inflammation. Heliyon. (2024) 10:e29686. doi: 10.1016/j.heliyon.2024.e29686. PMID: 38681642 PMC11046129

[40] Koning N van Eijk M Pouwels W Brouwer MSM Voehringer D Huitinga I . Expression of the inhibitory CD200 receptor is associated with alternative macrophage activation. J Innate Immun. (2010) 2:195–200. doi: 10.1159/000252803. PMID: 20375636

[41] Kotwica-Mojzych K Jodłowska-Jędrych B Mojzych M Kotwica-Mojzych K Jodłowska-Jędrych B Mojzych M . CD200:CD200R interactions and their importance in immunoregulation. Int J Mol Sci. (2021) 22(4):1602. doi: 10.3390/ijms22041602. PMID: 33562512 PMC7915401

[42] Zhao J Jiang L Deng L Xu W Cao Y Chen C . Important roles of CD32 in promoting suppression of IL-4 induced immune responses by a novel anti-IL-4Rα therapeutic antibody. MAbs. (2019) 11:837–47. doi: 10.1080/19420862.2019.1601985. PMID: 30950681 PMC6601543

[43] Ziegler-Heitbrock L . The CD14+ CD16+ blood monocytes: Their role in infection and inflammation. J Leukoc Biol. (2007) 81:584–92. doi: 10.1189/jlb.0806510. PMID: 17135573

[44] Bui TM Wiesolek HL Sumagin R . ICAM-1: A master regulator of cellular responses in inflammation, injury resolution, and tumorigenesis. J Leukoc Biol. (2020) 108:787–99. doi: 10.1002/JLB.2MR0220-549R. PMID: 32182390 PMC7977775

[45] Long EO . ICAM-1: Getting a grip on leukocyte adhesion. J Immunol. (2011) 186:5021–3. doi: 10.4049/jimmunol.1100646. PMID: 21505213 PMC3860744

[46] Jin Y Song Y Lin J Liu T Li G Lai B . Role of inflammation in neurological damage and regeneration following spinal cord injury and its therapeutic implications. Burns Trauma. (2023) 11:tkac054. doi: 10.1093/burnst/tkac054. PMID: 36873284 PMC9976751

[47] Ghosh M Lee J Burke AN Strong TA Sagen J Pearse DD . Sex dependent disparities in the central innate immune response after moderate spinal cord contusion in rat. Cells. (2024) 13:645. doi: 10.3390/cells13070645. PMID: 38607084 PMC11011714

[48] Hellenbrand DJ Quinn CM Piper ZJ Morehouse CN Fixel JA Hanna AS . Inflammation after spinal cord injury: A review of the critical timeline of signaling cues and cellular infiltration. J Neuroinflamm. (2021) 18:284. doi: 10.1186/s12974-021-02337-2. PMID: 34876174 PMC8653609

[49] Neirinckx V Coste C Franzen R Gothot A Rogister B Wislet S . Neutrophil contribution to spinal cord injury and repair. J Neuroinflamm. (2014) 11:150. doi: 10.1186/s12974-014-0150-2. PMID: 25163400 PMC4174328

[50] Kigerl KA McGaughy VM Popovich PG . Comparative analysis of lesion development and intraspinal inflammation in four strains of mice following spinal contusion injury. J Comp Neurol. (2006) 494:578–94. doi: 10.1002/cne.20827. PMID: 16374800 PMC2655318

[51] Milich LM Ryan CB Lee JK . The origin, fate, and contribution of macrophages to spinal cord injury pathology. Acta Neuropathol. (2019) 137:785–97. doi: 10.1007/s00401-019-01992-3. PMID: 30929040 PMC6510275

[52] Donnelly DJ Popovich PG . Inflammation and its role in neuroprotection, axonal regeneration and functional recovery after spinal cord injury. Exp Neurol. (2008) 209:378–88. doi: 10.1016/j.expneurol.2007.06.009. PMID: 17662717 PMC2692462

[53] Popovich PG Wei P Stokes BT . Cellular inflammatory response after spinal cord injury in Sprague-Dawley and Lewis rats. J Comp Neurol. (1997) 377:443–64. doi: 10.1002/(sici)1096-9861(19970120)377:3<443::aid-cne10>3.0.co;2-s. PMID: 8989657

[54] Schnell L Schneider R Berman MA Perry VH Schwab ME . Lymphocyte recruitment following spinal cord injury in mice is altered by prior viral exposure. Eur J Neurosci. (1997) 9:1000–7. doi: 10.1111/j.1460-9568.1997.tb01450.x. PMID: 9182952 PMC7163543

[55] Yee Mon KJ Goldsmith E Watson NB Wang J Smith NL Rudd BD . Differential sensitivity to IL-12 drives sex-specific differences in the CD8+ T cell response to infection. Immunohorizons. (2019) 3:121–32. doi: 10.4049/immunohorizons.1800066. PMID: 31317126 PMC6636834

[56] Balasubbramanian D Goodlett BL Mitchell BM . Is IL-12 pro-inflammatory or anti-inflammatory? Depends on the blood pressure. Cardiovasc Res. (2019) 115:998–9. doi: 10.1093/cvr/cvz028. PMID: 30698673

[57] Murphy CA Langrish CL Chen Y Blumenschein W McClanahan T Kastelein RA . Divergent pro- and antiinflammatory roles for IL-23 and IL-12 in joint autoimmune inflammation. J Exp Med. (2003) 198:1951–7. doi: 10.1084/jem.20030896. PMID: 14662908 PMC2194162

[58] Lago N Pannunzio B Amo-Aparicio J López-Vales R Peluffo H . CD200 modulates spinal cord injury neuroinflammation and outcome through CD200R1. Brain Behavior Immun. (2018) 73:416–26. doi: 10.1016/j.bbi.2018.06.002. PMID: 29870752

[59] Wynn TA Vannella KM . Macrophages in tissue repair, regeneration, and fibrosis. Immunity. (2016) 44:450–62. doi: 10.1016/j.immuni.2016.02.015. PMID: 26982353 PMC4794754

[60] Roved J Westerdahl H Hasselquist D . Sex differences in immune responses: Hormonal effects, antagonistic selection, and evolutionary consequences. Horm Behav. (2017) 88:95–105. doi: 10.1016/j.yhbeh.2016.11.017. PMID: 27956226

[61] Villa A Rizzi N Vegeto E Ciana P Maggi A . Estrogen accelerates the resolution of inflammation in macrophagic cells. Sci Rep. (2015) 5:15224. doi: 10.1038/srep15224. PMID: 26477569 PMC4609992

[62] Ferreira A Sousa Chambel S Avelino A Nascimento D Silva N Duarte Cruz C . Urinary dysfunction after spinal cord injury: Comparing outcomes after thoracic spinal transection and contusion in the rat. Neuroscience. (2024) 557:100–15. doi: 10.1016/j.neuroscience.2024.08.015. PMID: 39142624

[63] Guillot-Sestier MV Araiz AR Mela V Gaban AS O’Neill E Joshi L . Microglial metabolism is a pivotal factor in sexual dimorphism in Alzheimer’s disease. Commun Biol. (2021) 4:1–13. doi: 10.1038/s42003-021-02259-y. PMID: 34112929 PMC8192523

[64] Villa A Gelosa P Castiglioni L Cimino M Rizzi N Pepe G . Sex-specific features of microglia from adult mice. Cell Rep. (2018) 23:3501–11. doi: 10.1016/j.celrep.2018.05.048. PMID: 29924994 PMC6024879

[65] Moura M Miranda A Campos J Pinho AG Rito-Fernandes S Soares-Cunha C . Macro- and microanatomy of the sympathetic innervation of the spleen in rodents. J Comp Neurol. (2025) 533:e70086. doi: 10.1002/cne.70086. PMID: 40880187 PMC12396320

[66] Crane IJ Liversidge J . Mechanisms of leukocyte migration across the blood–retina barrier. Semin Immunopathol. (2008) 30:165–77. doi: 10.1007/s00281-008-0106-7. PMID: 18305941 PMC2315689

[67] Johnston B Butcher EC . Chemokines in rapid leukocyte adhesion triggering and migration. Semin Immunol. (2002) 14:83–92. doi: 10.1006/smim.2001.0345. PMID: 11978080

[68] Noor S Wilson EH . Role of C-C chemokine receptor type 7 and its ligands during neuroinflammation. J Neuroinflamm. (2012) 9:77. doi: 10.1186/1742-2094-9-77. PMID: 22533989 PMC3413568

[69] Song B Guo W He Y Yao X Sun J Wang S . Targeting immune cell migration as therapy for inflammatory disease: a review. Front Immunol. (2025) 16:1650760. doi: 10.3389/fimmu.2025.1650760. PMID: 41080585 PMC12507636

[70] Granger DN Senchenkova E . Leukocyte–endothelial cell adhesion. In: Inflammation and the microcirculation. San Rafael (CA): Morgan & Claypool Life Sciences (2010). Available online at: https://www.ncbi.nlm.nih.gov/books/NBK53380/. 21452440

[71] Hafezi-Moghadam A Thomas KL Prorock AJ Huo Y Ley K . L-selectin shedding regulates leukocyte recruitment. J Exp Med. (2001) 193:863–72. doi: 10.1084/jem.193.7.863. PMID: 11283159 PMC2193368

[72] Ivetic A Hoskins Green HL Hart SJ . L-selectin: A major regulator of leukocyte adhesion, migration and signaling. Front Immunol. (2019) 10:1068. doi: 10.3389/fimmu.2019.01068. PMID: 31139190 PMC6527602

[73] Ala A Dhillon A Hodgson H . Role of cell adhesion molecules in leukocyte recruitment in the liver and gut. Int J Exp Pathol. (2003) 84:1–16. doi: 10.1046/j.1365-2613.2003.00235.x. PMID: 12694483 PMC2517541

[74] Etzioni A . Adhesion molecules-their role in health and disease. Pediatr Res. (1996) 39:191–8. doi: 10.1203/00006450-199602000-00001. PMID: 8825786

[75] Reiss Y Engelhardt B . T cell interaction with ICAM-1-deficient endothelium *in vitro*: transendothelial migration of different T cell populations is mediated by endothelial ICAM-1 and ICAM-2. Int Immunol. (1999) 11:1527–39. doi: 10.1093/intimm/11.9.1527. PMID: 10464174

[76] Rothlein R Dustin ML Marlin SD Springer TA . A human intercellular adhesion molecule (ICAM-1) distinct from LFA-1. J Immunol. (1986) 137:1270–4. doi: 10.4049/jimmunol.137.4.1270 3525675

