# Supplementary material for: Sex differences in peripheral and local immune responses following spinal cord injury
Source: Front Immunol. 2026 May 25;17:1811925. doi: 10.3389/fimmu.2026.1811925 (PMC13243035; doi:10.3389/fimmu.2026.1811925)
Supplement: Supplementary Figure 1 — Flow cytometry gating strategy for blood sample staining. [file DataSheet1.docx]

Supplementary Material

# Supplementary Figures


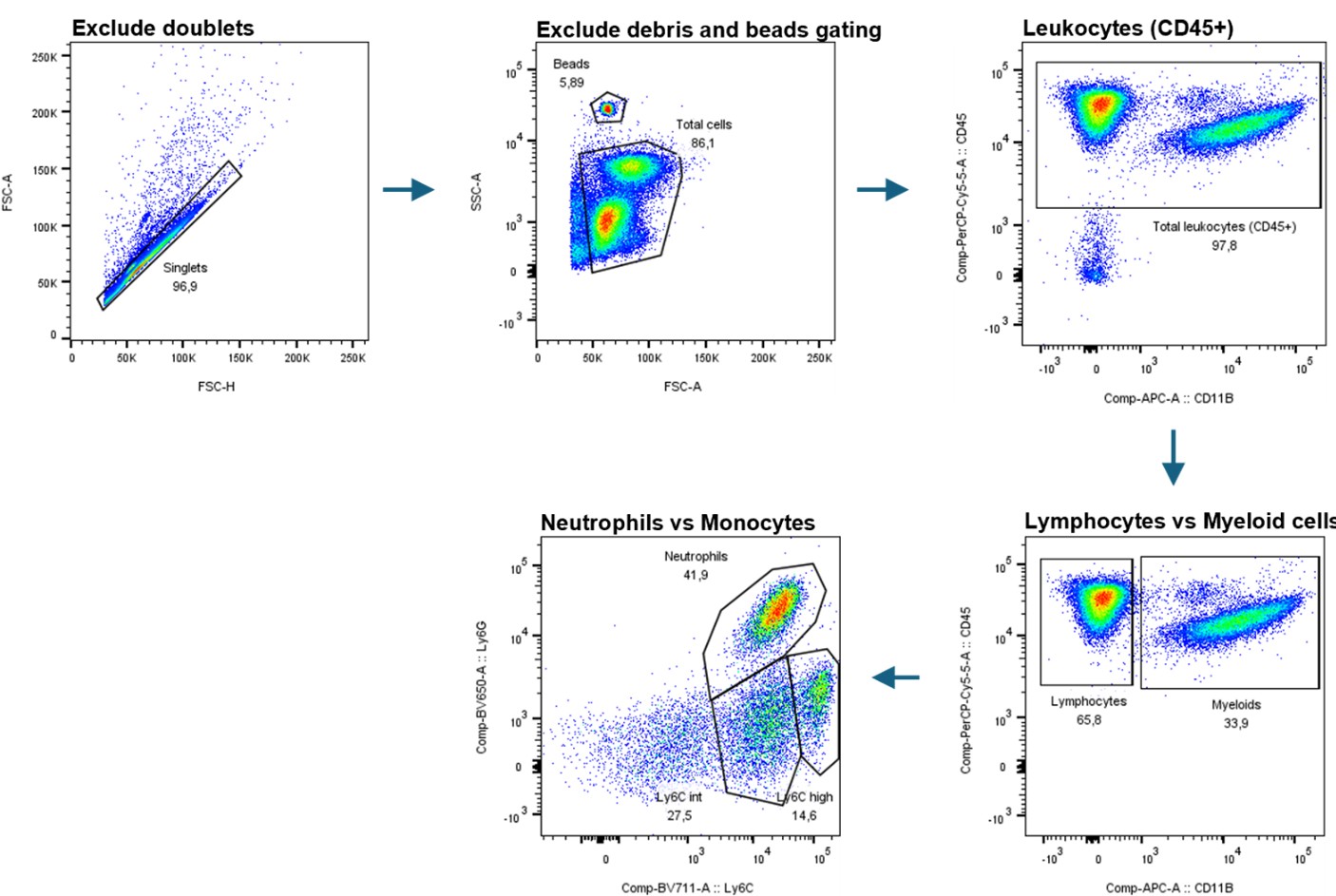


**Supplementary Figure 1.** **Fig. S1. Flow cytometry gating strategy for blood sample staining.**

**
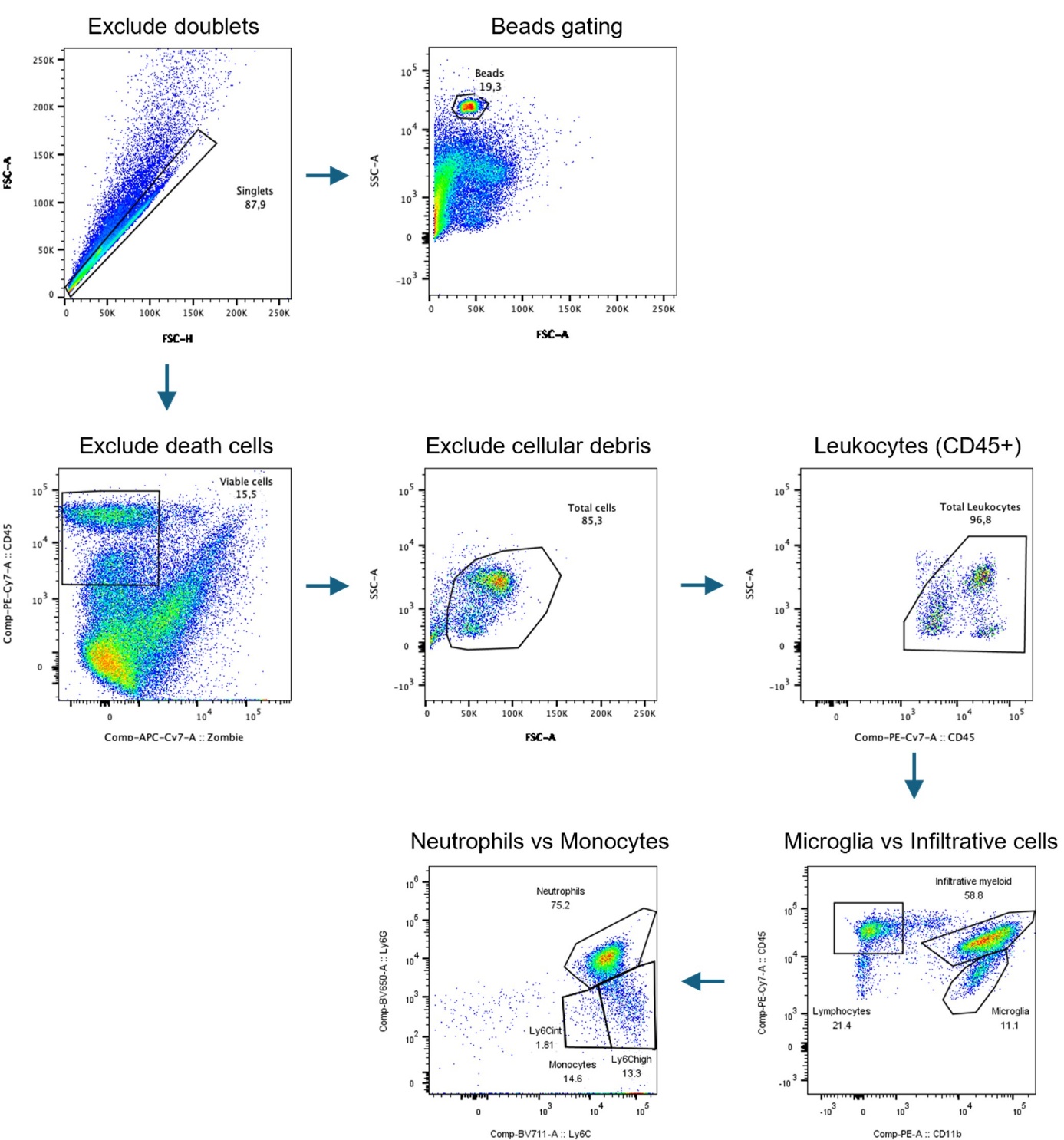
**

**Supplementary Figure 2.** **Fig. S2. Flow cytometry gating strategy for spinal cord single-cell suspension staining at 24 hpi.**

**
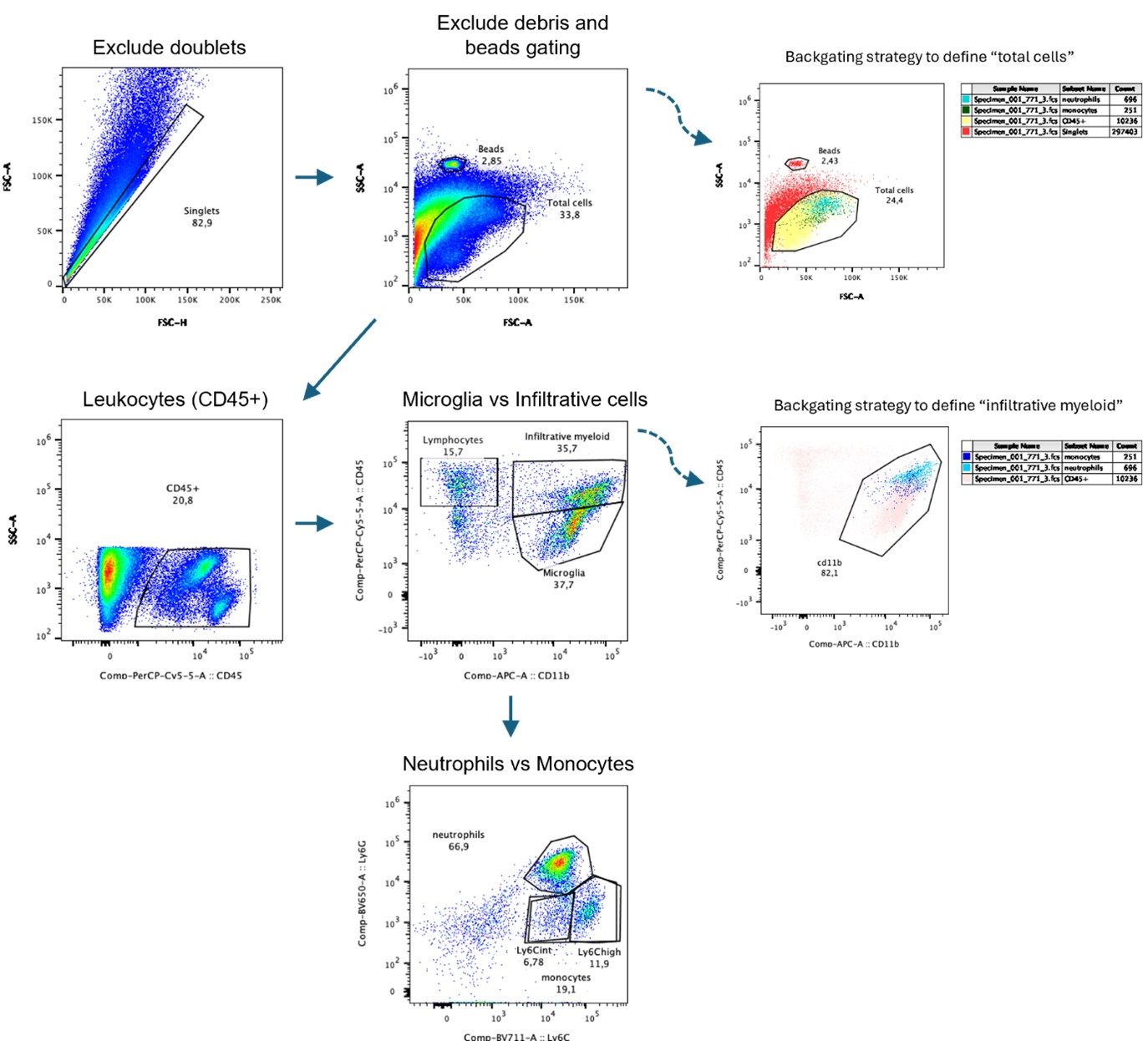
**

**Supplementary Figure 3.** **Fig. S3. Flow cytometry gating strategy for spinal cord single-cell suspension staining at 30 dpi.**

**
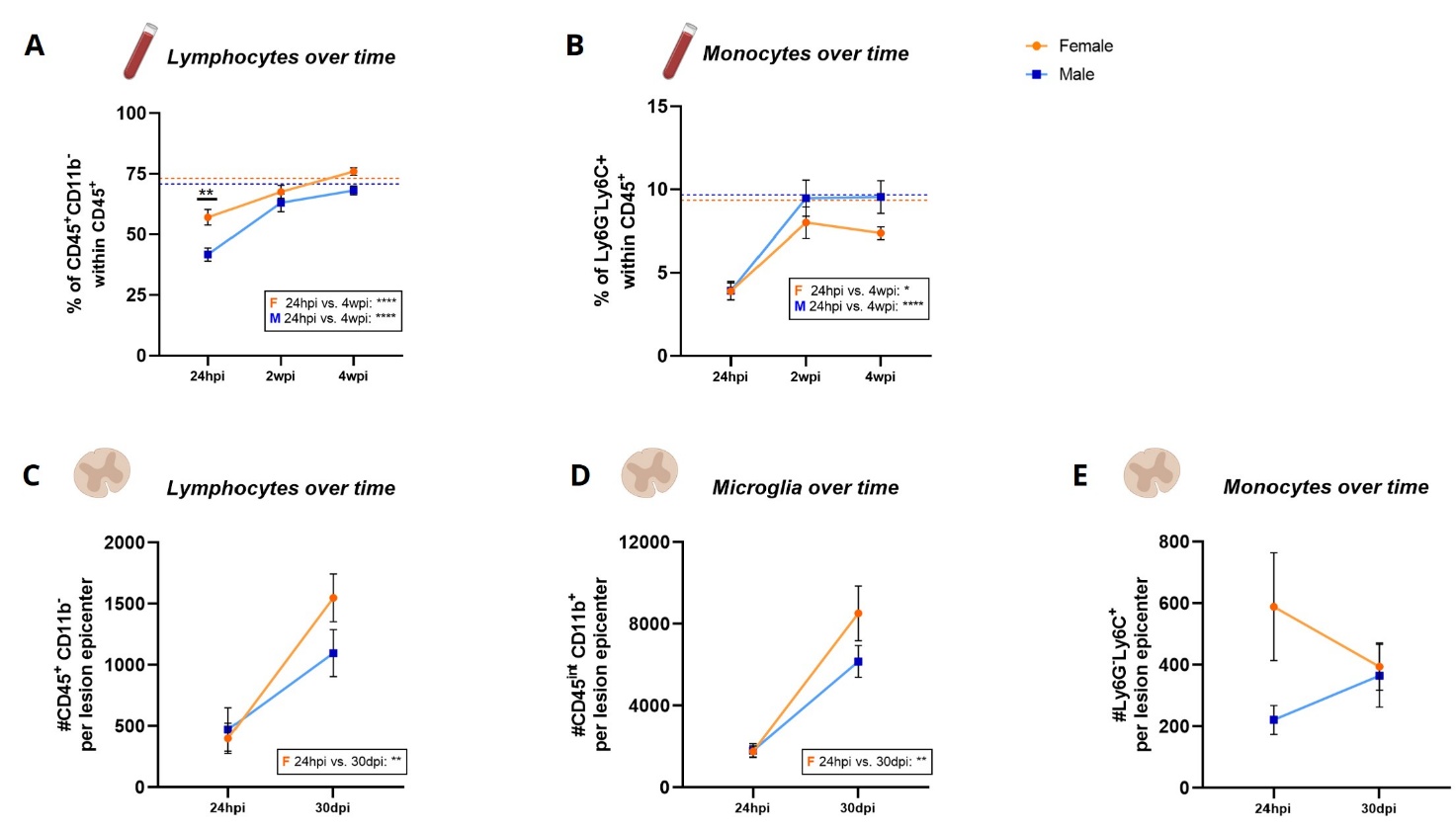
**

**Supplementary Figure 4.** **Fig. S4. (A-B)** Frequency of circulating **(A)** lymphocytes and **(B)** monocytes in the blood at 24 hours, 2 weeks, and 4 weeks post-SCI in male vs. female animals. **(C-E)** Total cell counts of **(C)** Lymphocytes, **(D)** Microglia and **(E)** monocytes in the spinal cord at 24 hours and 30 days post-SCI in male vs. female animals. Absolute cell numbers at the lesion epicenter were calculated using precision count beads. (Blood 24 hpi) n=13 for Female, n=11 for Male; (Blood 2 wpi) n=13 for Female, n=9 for Male; (Blood 4 wpi) n=11 for Female, n=9 for Male; (Spinal Cord 24 hpi) n=4 for Female, n=5 for Male; (Spinal Cord 30 dpi) n=9 for Female, n=6 for Male; Statistical tests: two-way ANOVA; post-hoc Tukey’s multiple comparisons test. Results expressed as mean ± SEM. *p ≤ 0.05, **p ≤ 0.01, ****p ≤ 0,0001.


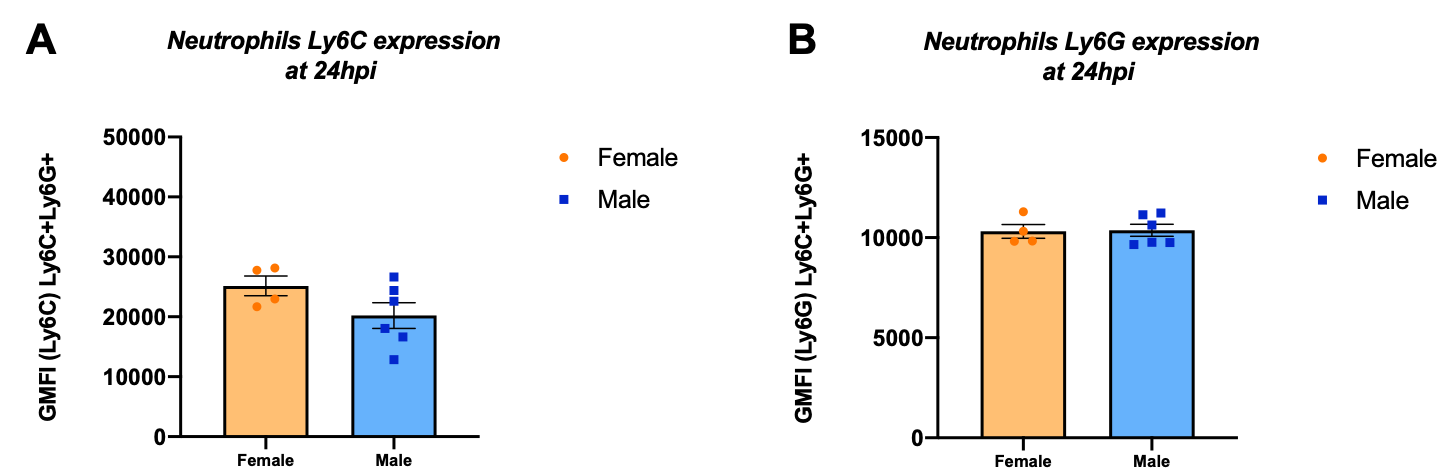


**Supplementary Figure 5. Fig. S5. (A-B)** Geometric mean fluorescence intensity (GMFI) of **(A)** Ly6C and **(B)** Ly6G expression in spinal cord-infiltrating neutrophils (Ly6G⁺Ly6C⁺) at 24 hours post-SCI in male vs. female animals. Quantification was performed at the lesion epicenter by flow cytometry. (Spinal Cord 24 hpi) n=4 for Female, n=5 for Male. Statistical tests: unpaired t-test. Results expressed as mean ± SEM

**
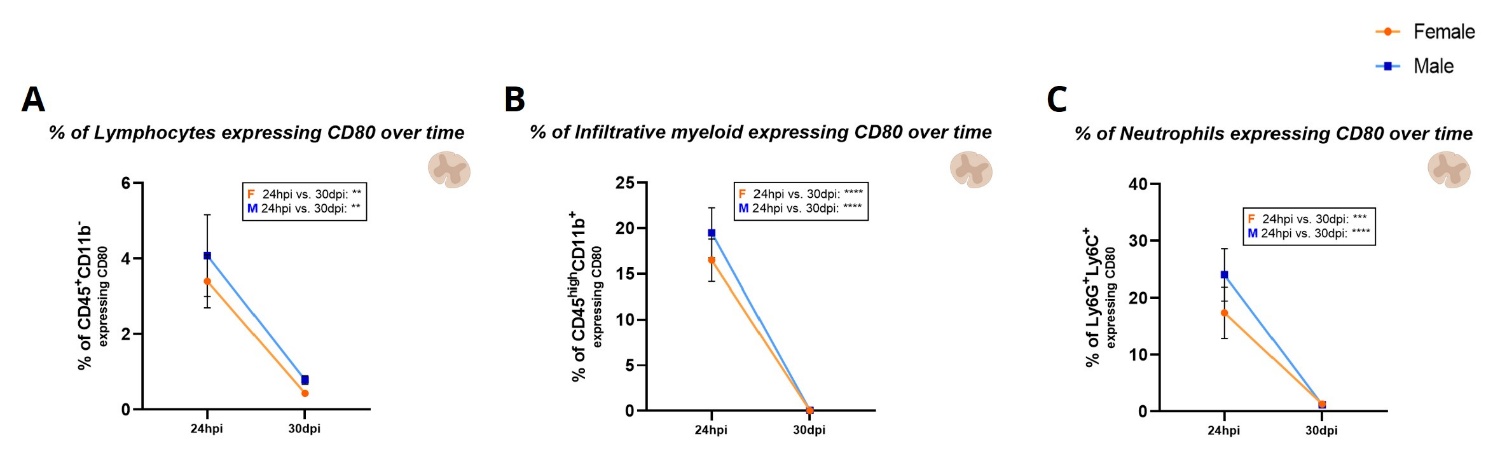
**

**Supplementary Figure 6.** **Fig. S6. (A-C)** Frequency of **(A)** lymphocytes, **(B)** infiltrative myeloid, and **(C)** neutrophils expressing CD80 in the spinal cord at 24 hours and 30 days post-SCI in male vs. female animals. (Spinal Cord 24 hpi) n=4 for Female, n=5 for Male; (Spinal Cord 30 dpi) n=9 for Female, n=6 for Male; Statistical tests: two-way ANOVA; post-hoc Tukey’s multiple comparisons test. Results expressed as mean ± SEM. **p ≤ 0.01, ***p ≤ 0.001, ****p ≤ 0,0001.


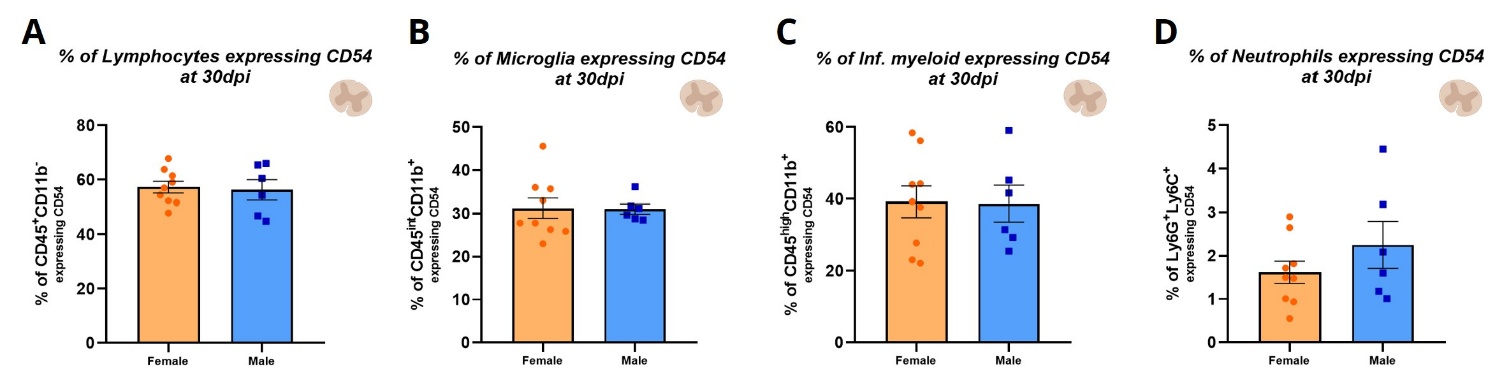


**Supplementary Figure 7.** **Fig. S7.** **(A-D)** Frequency of **(A)** lymphocytes, **(B)** microglia, **(C)** infiltrative myeloid, and **(D)** neutrophils expressing CD54 in the spinal cord at 30 days post-SCI in male vs. female animals. n=9 for Female, n=6 for Male; Statistical tests: Unpaired t-test. Results expressed as mean ± SEM.
